# Canonical and non-canonical roles of oligodendrocyte precursor cells in mental disorders

**DOI:** 10.1038/s44184-025-00133-x

**Published:** 2025-05-15

**Authors:** Giulia Poggi, Giulia Treccani, Martina von der Bey, Arnaud Tanti, Michael J. Schmeisser, Marianne Müller

**Affiliations:** 1 Institute of Anatomy, University Medical Center of the Johannes Gutenberg-University Mainz, Mainz, Germany; 2 Department of Psychiatry and Psychotherapy, University Medical Center of the Johannes Gutenberg-University Mainz, Mainz, Germany; 3 Department of Systemic Neuroscience Institute of Anatomy and Cell Biology, Philipps Universität Marburg, Marburg, Germany; 4 Molecular and Translational Neuroscience, Department of Neurology, University Hospital Ulm, Ulm, Germany; 5 Inserm, UMR 1253, iBrain, Université de Tours, Tours, France; 6 Focus Program Translational Neurosciences, University Medical Center of the Johannes Gutenberg-University Mainz, Mainz, Germany; 7 Leibniz Institute for Resilience Research, Mainz, Germany

**Keywords:** Diseases of the nervous system, Oligodendrocyte

## Abstract

Psychiatric research has shifted from a *neuroncentric* view to understanding mental disorders as disturbances of heterogeneous brain networks. Oligodendrocyte precursor cells (OPCs)— actively involved in the modulation of neuronal functions – are altered in psychiatric patients, but the extent and related consequences are unclear. This review explores canonical and non-canonical OPC-related pathways in schizophrenia, bipolar disorder, post-traumatic stress disorder, and depression in humans, highlighting potential mechanisms shared across diagnostic entities.

## The multi(dys)functional oligodendrocyte precursor cells

Mental illnesses impact on millions of people worldwide from any cultural background, gender and ethnic group. With a 25–30% lifetime prevalence worldwide^1^, mental illnesses are a severe global burden^2^ and impair daily functioning, relationships, and quality of life of both the affected individuals and their relatives. Despite the widespread prevalence, efficacious pharmacological interventions remain limited, and a large subset of patients does not respond adequately to the available treatments^3^. To overcome this challenge, it is essential to conclusively identify the cellular and molecular mechanism(s) implicated in the onset (or worsening) of the various mental disorders. In this regard, in the last few decades psychiatric research has departed from a *neuroncentric* view of mental (dys)functions and has dived towards a new perspective of psychiatry, which recognizes mental disorders as disturbances of brain networks and of the interplay between diverse cellular and molecular substrates. Emerging evidence suggests that, amongst other various biological substrates, also oligodendrocyte precursor cells might play a crucial role in the onset of mental disorders^4,5^.

Oligodendrocyte precursor cells (OPCs)—also referred to as oligodendrocyte progenitors or NG2-glia—are non-neuronal cells, homogeneously distributed within the central nervous system (CNS). They express specific markers, such as platelet-derived growth factor receptor alpha (PDGFRα) and NG2 proteoglycan, which distinguish them from mature oligodendrocytes and other glial cells (Fig. 2A)^6^. During mammalian development, OPCs arise from the ventral and dorsal embryonic spinal cord and from the ventral and dorsal forebrain^6^ and, in humans, develop also from the outer sub-ventricular zone^7^. A portion of OPCs differentiate into mature oligodendrocytes already at early developmental stages, while other OPCs maintain the progenitor state, including the capacity of migrating and proliferating, and reside in the CNS into and throughout adulthood^6,8^. Conventionally, these resident OPCs act as the reservoir of progenitor cells that can differentiate into oligodendrocytes and sustain myelination^8,9^. However, it is worth mentioning that a profound degree of heterogeneity has been observed in the rate of proliferation and differentiation and in the cell cycle duration of these NG2-glia, especially when comparing grey matter and white matter^10^. This is generally referred to as the myelination-related *canonical role* of OPCs^11^. This well-established canonical role is fundamental for maintaining neural health and functionality, providing metabolic support to the axons and ensuring saltatory conduction^12,13^. Intriguingly, in the last few years preclinical studies have disclosed multiple additional myelination-independent functions of OPCs within the brain, including their involvement in synaptic regulation, response to injury, immune modulation, maintenance of blood brain barrier integrity and extracellular K^+^ homeostasis, and neuronal plasticity/activity^8,14^. These are typically referred to as myelination-unrelated *non-canonical roles* of OPCs^11^.

OPCs are extremely responsive to changes in the *molecular milieu* in their surroundings and they can proliferate, migrate, differentiate and initiate myelination in response to stimuli, including neuronal activity^14–19^. Also, OPCs receive direct synaptic inputs from GABAergic and glutamatergic neurons^17,20,21^ and recent evidence suggest that OPCs can synapse onto GABAergic neurons, directly regulating their functions^22^. Importantly, OPCs and oligodendrogenesis have been implicated in the accomplishment of motor, somatosensory and cognitive tasks^23–28^, further highlighting their diversified roles in brain functions. Conceivably in virtue of this functional heterogeneity, this responsiveness to stimuli and this crucial role in the accomplishment of behavioural tasks, OPCs have increasingly gained visibility in the field of psychiatry, and recent lines of evidence support the implication of OPCs (dys)function in mental illnesses (see e.g^5,29,30^.). However, a comprehensive overview of the current knowledge about OPCs implication in psychiatry, specifically in human samples, is missing. Thus, the current review aims at providing a comprehensive and up-to-date overview of the OPCs-related clinical findings in the context of mental illnesses. The identification of potential converging role of OPCs-related pathological (canonical and non-canonical) pathways and the detection of knowledge gaps would provide an essential timely insight to instruct future studies and, possibly, pave the way for the development of new and more efficacious therapeutic strategies for mental illnesses or for the implementation of preventive measures to preserve mental health.

## Oligodendrocyte precursor cells in psychiatric diseases

### Screening procedure and bibliometric assessment

**Fig. 1:**
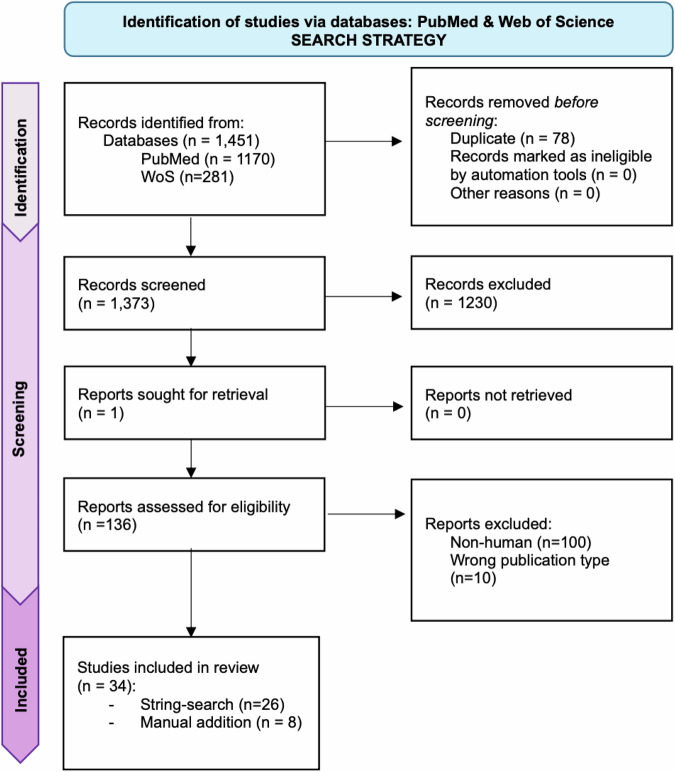
Search strategy. Flow diagram of the searches of databases for the selection of the relevant research articles. The diagram reports the step-by-step screening of the relevant literature that was retrieved on PubMed (https://pubmed.ncbi.nlm.nih.gov/) and Web of Science (https://www.webofknowledge.com) with the reported search strings (see Supplementary Note 1). The diagram was adapted from the PRISMA guidelines for systematic review^31^.

**Fig. 2:**
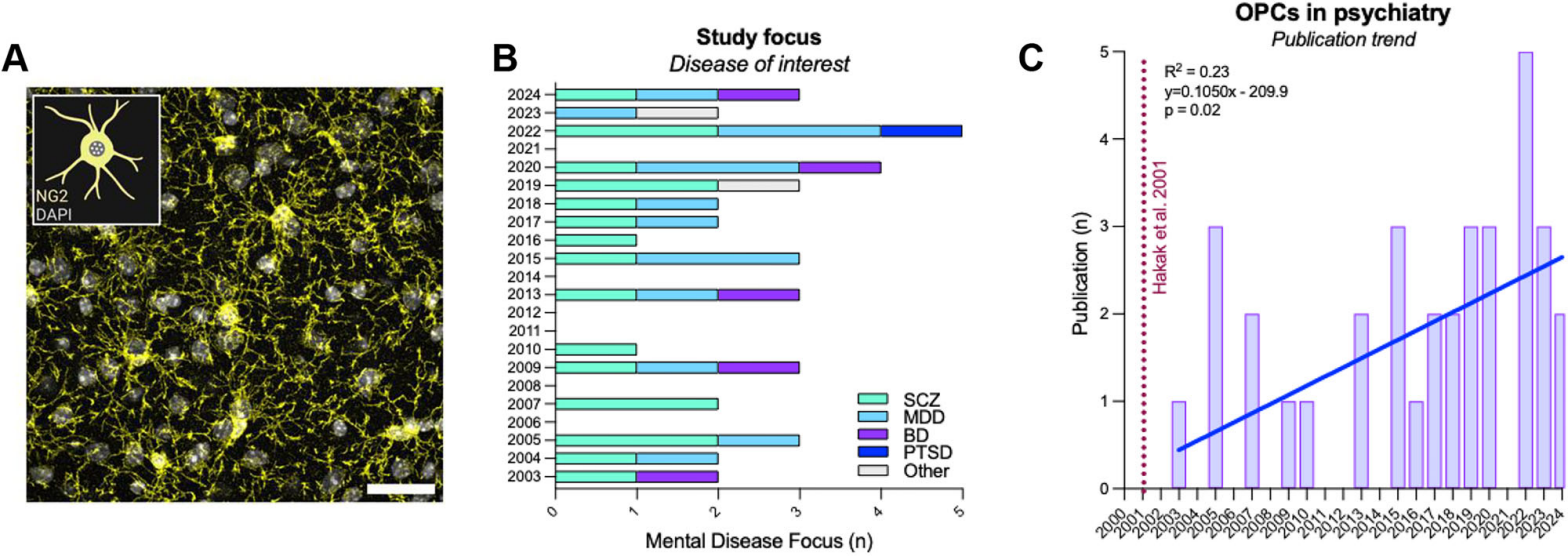
Biometric analysis of the research articles published on the implication of OPCs in the onset of mental disorders. **A** Representative confocal micrograph showing NG2-glia in the amygdala of an adult male C57Bl6/j mouse. Scale bar = 20 μm. **B** Graphical representation of the disease focus of the research articles included in this review (number of studies) plotted against the year of publication (SCZ = schizophrenia; MDD = major depressive disorder; BD = bipolar disorder; PTSD = post-traumatic stress disorder; Other = any mental disease other than SCZ, MDD, BD and PTSD). **C** Publication trend with respect to research articles investigating OPCs in psychiatry. Linear regression shows a mild but significant trend for an increase in the number of studies focusing on OPCs in mental illnesses related to the year of publication (R2 = 0.23, *p* = 0.02).

The screening of two databases (Web of Science and PubMed) retrieved a total of 1960 publications (see search strings in Supplementary Note 1), which was then filtered according to relevance, publication type, and species (i.e. human). A total of 26 research articles were identified via the reported search strings (see Supplementary Note 1) and 8 additional research articles were manually included. The latter were identified amongst the references of the articles retrieved through the search strings. A comprehensive flow diagram of the searches, adapted from the PRISMA guidelines for systematic review^31^, is reported Fig. 1. Overall, we included a total of 34 research articles in the English language that evaluated OPCs changes in the context of mental disorders and that were published between 2003 and 2024. Although the search strings allowed for the detection of a broad range of psychiatric illnesses, the included studies focused on a small sub-group of disorders, namely on *schizophrenia* (SCZ, *n* = 18)^32–49^, *major depressive disorder* (MDD, *n* = 14)^36,39,46,50–60^, *bipolar disorder* (BD, *n* = 5)^32,36,39,46,49^, and *post-traumatic stress disorder* (PTSD, *n* = 1)^61^. Some of the studies included more than one disease group^32,36,39,46,49^ and two studies combined symptoms of multiple mental disorders and/or did not use the conventional diagnostic categories^62,63^. A timeline of the disease focus of the included articles is provided in Fig. 2B. In most of the studies, the authors recruited (or employed samples from) females and males, except for 4 studies that were carried out exclusively in male samples^44,53–55^, with two of the latter published by the same group^53,54^. Most of the included post-mortem studies focused on cortical and sub-cortical regions, and limbic areas relevant to cognitive and emotional functions that are usually compromised in psychiatric patients. A variety of methodological approaches was employed, including immunohistochemistry, immunoblotting, mRNA level quantification (qPCR, microarray, bulk-RNAseq, single-nucleus RNAseq, and single-cell RNAseq), proteomics, iPSC-derived cells, SNPs genotyping, and computational methods to re-analysed already published RNA-sequencing datasets. With respect to the latter, the RNA-seq dataset in MDD patients provided by Nagy et al. ^54^ was one of the most re-employed databases within the selected studies. With respect to the examined OPCs-related pathways, 4 studies in MDD^51,54,56,58^ and 1 study in SCZ^48^ reported on findings that were directly related to the non-canonical pathways of the OPCs, while the remaining findings were mostly related to the conventional myelinogenic role of OPCs. Critically, the first publication addressing the role of non-canonical mechanisms of OPCs in psychiatric illnesses was published in 2015 and most of the discoveries on non-canonical OPCs pathway since then were published by the same research group and/or originated from the same dataset. This draw attention on the extreme need of additional independent studies to confirm (or disprove) these findings. Accordingly, the publication trend (Fig. 2C) shows that the field is slowing but steadily growing, with the number of studies focusing on the role of OPCs in psychiatry in constant increase. A comprehensive summary of the included articles is reported in Supplementary Table 1 and Supplementary Table 2.

**Fig. 3:**
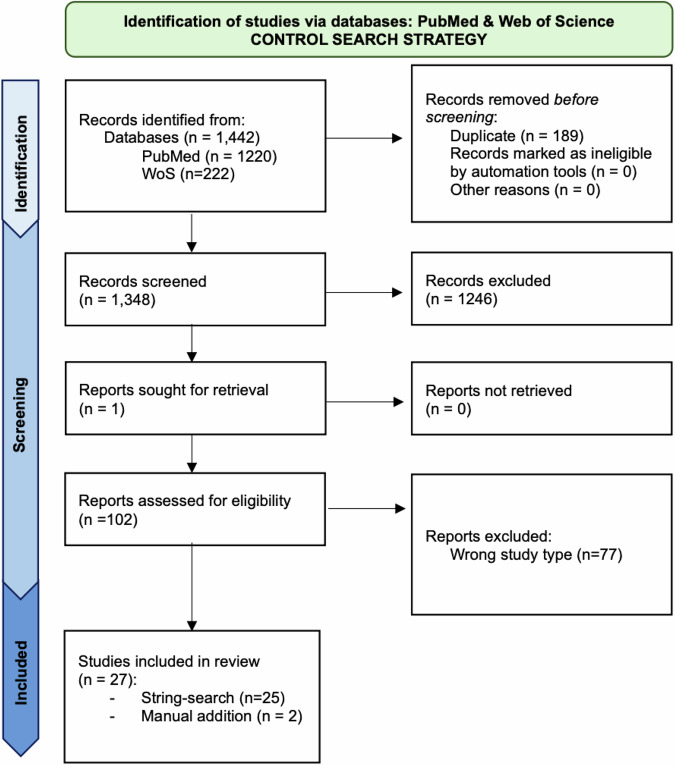
Control search strategy. Flow diagram of the searches of databases for the selection of the relevant research articles. The diagram reports the step-by-step screening of the relevant literature that was retrieved on PubMed (https://pubmed.ncbi.nlm.nih.gov/) and Web of Science (https://www.webofknowledge.com) with the reported control search strings (see Supplementary Note 2). The diagram was adapted from the PRISMA guidelines for systematic review^31^.

The search strings were designed with the specific goal of retrieving every study that explored the contribution of OPCs (dys)function to the onset (or worsening) of the indicated psychiatric pathologies. Yet, this approach alone could lead to overestimation of the involvement of OPCs in the pathophysiology of mental illnesses. To control for this, we designed additional search strings to contextualise OPC-related findings within published comprehensive and unbiased cell type analyses performed in post-mortem brains of psychiatric patients. We limited our search strategy to studies that employed single-cell RNA sequencing and single-nucleus RNA sequencing to compare control individuals with psychiatric illnesses (see search strings in Supplementary Note 2). Single-cell multiomics analysis employing BICCN or PsyENCODE scRNAseq were also included. A comprehensive flow diagram of the searches, adapted from the PRISMA guidelines for systematic review^31^, is reported in Fig. 3.

### OPCs alterations in severe mental illnesses

For decades, alterations in the oligodendrocyte lineage and myelin have been observed in individuals affected by mental illnesses and/or neurodevelopmental and neurodegenerative diseases (see e.g^64–66^.). Yet, it is with the beginning of the 21^st^ century and the implementation of high-throughput unbiased screenings that the field of psychiatry has abandoned the dogma of “*brain dysfunction equal neuron dysfunction*” and has started diving into the role of the oligodendrocyte lineage in the onset (or worsening) of mental disorders. The work from Hakak and co-authors^67^, unbiasedly showing a reduction of oligodendrocyte-related transcripts in the dorsolateral prefrontal cortex of SCZ individuals as compared with matched controls, is possibly the symbol of such conceptual revolution in the field of schizophrenia research (Fig. 2C). Probably inspired by this pioneering discovery, other groups started exploring additional oligodendrocyte lineage-related markers in independent cohorts of SCZ individuals and in individuals affected by other psychiatric pathologies, including MDD and BD (see e.g^68–70^.). Of relevance to the topic of the current review, the first findings on OPCs in psychiatric illnesses go back to 2003, when Tkachev and colleagues quantified transcriptional changes in the Brodmann area (BA) 9 of SCZ and BD post-mortem samples via microarray technology^32^. Although OPCs were not the main and only focus, this work could be considered the first human study that quantified OPCs-specific markers in post-mortem psychiatric samples. In the following sub-sections, we will discuss OPCs-specific findings, according to conventional diagnostic categories.

#### Schizophrenia

Schizophrenia is a severe polygenic mental disorder with a 1% lifetime prevalence worldwide and with a slightly higher incidence in males than in females. Symptoms of SCZ generally begin in late adolescence or early adulthood and include hallucinations, delusions, blunted emotions and cognitive dysfunctions^71,72^. The aetiology of SCZ is still unclear. The occurrence and the severity of the symptoms can vary substantially between patients and multiple cellular and molecular substrates have been proposed to contribute to the onset of the disease, including mature oligodendrocytes and myelin^73^. Although to a lesser extent, also OPCs have been implicated in the occurrence of SCZ. In the following sub-sections, we provide an overview of: (1) genetic mutations and/or variations (in some instances corroborated via hiPSC technology), (2) gene expression, and (3) histological alterations related to OPCs pathology in SCZ.

##### Genetic variations and hiPSC-based findings

Studies on rare and common genetic variations have highlighted the possible contribution of OPCs dysfunction to the onset of SCZ. A genome-wide linkage disequilibrium study in a cohort of 175 family with at least two siblings affected by SCZ (or other psychotic disorders) showed a linkage at the Chr15q22-24, which contains the gene coding for chondroitin sulphate proteoglycan 4 (CSPG4)/NG2^74,75^. Yet, it is worth mentioning that this finding has not been replicated. Also, it has been reported that multiple rare missense mutations in the gene coding for CSPG4/NG2 segregate with SCZ and that carriers of the rare CSPG4^A131T^ mutation are affected by SCZ or present with history of psychiatric illnesses^44^. Critically, iPSCs-derived neurons from individuals carrying this specific mutation do not differ from iPSCs-derived neurons of healthy controls, except for minor dissimilarities in input resistance and action potential threshold. Whilst neurons appeared to be rather unaffected, the iPSCs-derived OPCs presented a variety of alterations^44^. The latter included: reduced cell size, aberrant post-translational processing of the NG2 protein and related protein folding deficits, as well as abnormally increased co-localization of NG2 protein with the endoplasmic reticulum marker calreticulin, which highlighted a profound alteration in subcellular localization^44^. Such changes were paralleled by a general reduction of oligodendrogenesis and cell viability^44^. Interestingly, these same SCZ CSPG4^A131T^ carriers showed a reduction in global fractional anisotropy (FA) and increased microstructural alterations in the white matter, robust indication of myelin deficits. This suggest that the oligodendrocyte lineage, rather than neurons, might play a central role in the aetiology of this form of familial SCZ^44^. In another study employing iPSC technology, it was observed that iPSCs derived from SCZ produced a significant lower number of O4^+^-Oligodendrocyte lineage cells compared to healthy controls. Importantly, in this study none of the patients was a carrier for any CSPG4 rare variants, pointing out that the implication of OPCs in SCZ is not restricted to rare forms of the disease^43^. Also, a positive correlation between the percentage of O4^+^ cells and Magnetization Transfer Ratio (MTR) – conventional MRI readout for myelin content—in frontal white matter was observed in SCZ, but not in controls, suggesting that the delay in OPCs differentiation observed in vitro might be the source of deficits in white matter content in patient, specifically^43^.

Another study on familial SCZ provided further evidence for the involvement of the oligodendrocyte lineage in SCZ. Specifically, Windrem and colleagues^42^ generated bipotential astrocyte-oligodendrocyte human glial progenitor cells (hGPCs) from patient-specific human iPSCs and transplanted them into the brain of *Shiverer* mice, which lack myelin basic protein (MBP) and CNS myelin^76^. When comparing the colonization pattern, it became apparent that some of the SCZ-derived hGPCs showed abnormal migration capability and were unable to colonize the mouse white and grey matter as efficiently as the control-derived hGPCs. This was paralleled by hypomyelination and downregulation of synaptic markers. Based on the latter, the authors proposed that the deficits in brain colonization by hGPCs might have been mediated by dysfunction in neuron-glia synaptic communication^42^. Of note, such deficits were not detected in every SCZ-derived hGPCs included in the study, further highlighting the well-established complexity and heterogeneity of this pathology^42^.

Supporting the implication of OPCs in the occurrence SCZ independently of rare genetic variants, Papiol et al. ^45^ showed that polygenic risk score (PRS) for OPCs, but not for mature oligodendrocyte, was associated with volume changes in the left CA4/Dentate Gyrus of the hippocampus of SCZ patients exposed to aerobic exercise. Specifically, higher OPC-associated genetic risk burden was coupled with lower increase of CA4/Dentate Gyrus volume. This suggested that the beneficial effect of high intense exercise is related to cell type-specific polygenic risk score, which might impact on hippocampal plasticity of a sub-group of SCZ patients and determine their responsiveness to certain non-pharmacological interventions^45^. In another study combining gene, gene expression and brain morphometry, it was proposed that heterogeneity in regional cortical thickness in SCZ is mediated by a cell specific genetic predisposition and it was shown that OPC-related genetic risk load was associated with a less severe cortical thinning^47^. Although these findings require further validation, they draw additional attention on the heterogeneity of SCZ^47^.

##### Gene expression and protein alterations

The heterogeneity that emerges from the genetic findings is also reflected in post-mortem studies. With respect to gene expression, it was reported that the level of CPSG4/NG2 transcripts was increased in the putamen^36^ and unchanged in the BA9^32^ of SCZ individuals as compared to matched controls. Furthermore, two additional studies reported a reduction of *OLIG1*, *OLIG2* and *SOX10* transcripts in multiple cortical regions – including BA9 – of SCZ as compared with matched controls^32,33^ and one study identified high methylation at the *SOX10* DNA region that is associated with reduction of *SOX10* expression in SCZ patients^77^. Conceivably, these changes pertained the mature myelinating oligodendrocytes, rather than the OPCs, since *NG2* and *PDGFRA* mRNA was unaltered and, in contrast, multiple myelin-related genes were reduced^32,33^. However, it is important to point out that other studies could not fully replicate these findings^34,35^ and proposed that some of the oligodendrocyte lineage-related transcriptional changes might be influenced by genetic background and individual predisposition^34^ or exclusively mediated by antipsychotic medications^35^.

Further studies detected changes in transcripts and proteins that are fundamental for OPC functions, although not solely expressed in OPCs, drawing attention on potential changes in OPCs migration and proliferation/maturation dynamics in SCZ. Specifically, through state-of-the-art, bottom-up shotgun mass spectrometry, Saia-Cereda et al. detected alterations in the Ephrin B signalling pathway – including guanine nucleotide binding protein (GNB4), and vav guanine nucleotide exchange factor 2 (VAV2) – in the corpus callosum of SCZ samples as compared to matched controls^41^, and suggested that this might be related to dysfunction in neuron-OPC communications, which ultimately could impact OPCs migration^41^. It should be noted though that these markers are highly expressed in microglia, astrocyte and neurons and somewhat less enriched in the oligodendrocyte lineage, therefore it cannot be excluded that dysfunctions in this pathway might have more severe effects on cell types other than OPCs^78^. Also, with a computational approach that combined multiple available bulk-RNAseq datasets, Aranda and colleagues reported that specific isoforms of Discoidin Domain Receptor Tyrosine Kinase 1 (*DDR1*) transcripts, known to be enriched in OPCs and to be related to cell morphology changes during cell cycle, are reduced in the dorsolateral prefrontal cortex of SCZ patients as compared to matched controls^49^. Furthermore, Kerns and colleagues identified a correlation between OPC markers and cell cycle-related transcripts in the internal capsule of SCZ patients and suggested a deceleration in the turnover of the oligodendrocyte OL lineage in SCZ as compared to controls^37^. Whilst somewhat in line with the findings from Aranda and colleagues, hinting at alterations in OPC cell cycle and proliferation-maturation dynamics in SCZ, it should be noted that postmortem interval (PMI) and age of SCZ samples differ substantially from the control samples employed by Kerns and colleagues^37^. Thus, caution should be taken in the interpretation of these findings, as these technical differences might have an impact on the reported readouts.

##### Histological alterations

With respect to histological investigations, Kolomeets and colleagues performed Nissl staining on post-mortem brains from SCZ and matched controls and quantified the density of OPCs (identified as oligodendrocyte cluster) and mature oligodendrocyte in the parietal cortex – BA 39, BA40 – and in the putamen^38,46^. They showed that adolescent onset SCZ individuals presented with a reduction of OPCs density in layer 3 of BA39 and of BA40, while adult onset SCZ individuals displayed a reduction of OPCs density only in layer 3 of BA39^38^. Additionally, they reported a reduction in OPC density in the putamen of male SCZ, but not in females^46^. In contrast, independent studies that investigated the frontal cortex did not detect any change in OPC density in SCZ. Specifically, Mosebach et al. ^39^ took advantage of the cellular distribution of the marker OLIG1 to distinguish OPCs from mature oligodendrocytes, claiming that OLIG1 is mostly expressed in the nuclear compartment of OPCs and is preferentially located in the cytoplasm of mature oligodendrocytes. With this approach, the authors detected comparable density of OPCs in the pregenual anterior cingulate (BA32), in the dorsolateral prefrontal cortex (BA9), and in the adjacent white matter of SCZ vs. controls^39^. Consistently, Mauney et al. ^40^, employing CSPG4/NG2 marker in an independent post-mortem cohort, reported that the density of OPCs in BA9 was unchanged in SCZ as compared with matched controls. Whilst the alteration of OPC density does not seem to be a feature of the frontal cortex^39,40,48^, an independent study reported that OPCs in the frontal cortex of SCZ displayed a significant increase in their morphological complexity. Additionally, through the implementation of preclinical models, the authors proposed in the same study that OPCs hypertrophy might be mediated by an increased expression of the truncated Δ3 and Δ7 isoforms of *Disrupted in schizophrenia 1 (DISC1)*^48^, a well-established candidate risk gene for SCZ^79^. Whether OPCs hypertrophy would precede OPCs death and ultimately result in reduced density of OPCs, mimicking the endpoint observed in parietal regions of the brain, or it is a genuine brain region-specific pathological response that does not entail OPCs loss remains an open question.

Overall, the published studies disclose a significant degree of heterogeneity with respect to OPCs alterations in SCZ and raise the intriguing question as to whether OPCs pathology could pertain exclusively a sub-group of SCZ patients who might present with a well-defined and specific subset of symptoms. Importantly, when OPCs alterations are detected in this subset of patients, they appear to mostly impact on the canonical myelinogenic pathway.

#### Bipolar disorder (BD)

Bipolar disorder (BD), also identified as manic depression, is a severe chronic mood disorder characterized by alternating episode of depression, mania and hypomania. Based on its longitudinal course and on the occurrence of these specific episodes, BD can be classified in BD type I (when at least one manic episode occurs) and BD type II (when at least one hypomanic and one depressive episode occur)^72,80^. Individuals that present with hypomanic and depressive symptoms, but do not fulfil the criteria for either BD type I or BD type II are assigned to a separate diagnostic category, namely cyclothymic disorder^72,80^. The lifetime prevalence worldwide for the overall BD spectrum is estimated to be 2.4%^80^. The aetiology of BD is currently unknown, but environmental and genetic factors have been implicated in the onset of the disease, and, as well as for SCZ, findings on the pathophysiology of BD are quite heterogeneous and include changes in oligodendrocyte lineage and white matter integrity (for review see refs. ^81,82^).

With respect to OPCs, the findings are quite limited and mostly overlapping with what is observed in SCZ patients, suggesting that OPC dysfunction might mediate psychiatric symptoms common to both diseases, such as psychotic symptoms. Specifically, through the integration of multiple bulk-RNAseq datasets, Aranda and colleagues reported that OPC-enriched isoforms of Discoidin Domain Receptor Tyrosine Kinase 1 (*DDR1*) are reduced in the dorsolateral prefrontal cortex of BD patients as compared to matched controls^49^. Furthermore, and again in line with findings in SCZ, it was observed that in the putamen of BD individuals the level of *CPSG4/NG2* transcripts was increased for males and females^36^, but, in a different study, the density of OPCs was reduced only in males affected by BD as compared to matched controls^46^. With respect to cortical regions, including BA32 and BA9 and adjacent white matter, the density of OPCs was unchanged in BD as compared to controls^39^. As well as for SCZ, most OPCs-related findings in BD have been evaluated in the context of the canonical myelinogenic pathway.

#### Major depressive disorder (MDD)

Major depressive disorder (MDD) is a serious mood disorder, with a 15–18% lifetime prevalence worldwide, and almost twice as common in women than in males^72,83^. Symptoms of MDD can appear at any time from adolescence to late adulthood and consist of depressed mood, anhedonia, feelings of worthless and/or guilt, thought of death, suicidal ideation, and appetite and sleep disturbances^72^. Multiple biological systems have been implicated in the onset (or exacerbation) of MDD, and aversive life experiences and/or psychosocial stressors have been shown to increase the likelihood of developing MDD^84,85^.

A few studies have explored the role of OPCs in MDD, employing microarray technology, RNAseq and histological approaches.

##### Gene expression alterations

Through microarray technology, Aston et al. ^50^. performed a gene expression analysis on the temporal cortex of MDD postmortem samples compared to matched controls. They identified transcriptional changes in various biological pathways, including neurodevelopment, signal transduction, cell communication and myelination. With respect to the latter and of relevance for this review, they detected a reduction of *SOX10* and *OLIG2* mRNA, both pan-markers for the oligodendrocyte lineage, and a reduction of additional myelin-related transcripts^50^. These findings suggest that the implication of OPCs in MDD was likely to be related to oligodendrocytes and the canonical myelinogenic pathway^50^.

A single nucleus transcriptomic study carried out on male suicide completers affected by MDD compared to controls drew attention on a new perspective on OPCs in MDD and, in more general terms, in psychiatry. Indeed, this study revealed that almost 50% of the detected transcript dysregulation in BA9 of MDD postmortem samples pertained two cellular clusters, namely deep layer excitatory neurons and immature OPCs^54^. Critically, through a computational exploratory approach, the authors identified significant changes in number of ligand-receptor combinations between these two cellular clusters, hinting at deficits in neuron-OPCs communications and at the involvement of non-canonical OPCs pathway in the onset of MDD^54^. This dataset was employed for additional computational studies, either alone^55,57,59^ or in combination with other RNA datasets^57,58^. A brief summary of the main findings derived from this database is provided in the following paragraph.

Sex-specific analysis of the dataset by Nagy et al. ^54^ in combination with an additional dataset including female samples confirmed that clusters of transcripts related to astrocytes, OPCs and excitatory neurons are mostly altered in males affected by MDD, while clusters of transcripts related to parvalbumin interneuron and microglia are mostly altered in females^57^. Xie and colleagues^59^ defined four distinct oligodendrocyte lineage developmental stages based on gene expression and identified markers that are either relevant to a specific developmental stage or that promote stage transition. Through this approach, they showed that OPC-gene clusters exhibit the best predictive ability for the occurrence of MDD, underlining the relevance of this cell type in the MDD pathology. Moreover, again with the same dataset^54,59^, Kokkosis et al. detected a new MDD-specific subtype of oligodendrocytes, namely immune-oligodendrocytes. According to complementary preclinical data, immune oligodendrocytes might regulate microglia activity and its downstream effects on myelin integrity^55^. Lastly, Zhou et al. combined bulk-RNAseq, single nucleus RNAseq and/with DNA methylation datasets from MDD vs. matched controls [ref: GSE102556, GSE88890, GSE144136, and GSE197622^54,86–88^] and identified major changes the ion channel and glutamate receptor pathways. Importantly, such alterations were enriched in OPCs^58^. Whilst the current evidence supports the implication of OPCs alterations in the pathophysiology of MDD in this specific cohort, additional replication studies – employing a different human cohort and including both genders – would be essential to further confirm the findings originated from the dataset of Nagy and colleagues^54^.

##### Histological alterations

With respect to histological studies, reduced OPC density was detected in the putamen of males affected by MDD, while mature oligodendrocyte density was unaltered^46^. However, in an independent study, *CSPG4/NG2* mRNA level was unchanged in the putamen of MDD vs controls^36^. This might suggest that the extant OPCs in MDD express a higher level of *NG2* as compared to controls. Intriguingly, in preclinical models it has been shown that NG2 can be cleaved and released in the extracellular compartments to maintain physiological neuronal functions and related behavioural skills^14^. Thus, a putative increase of NG2 level in the extant OPCs in the putamen of MDD might be a compensatory mechanism that acts through this non-canonical OPCs pathway influencing neuronal activity.

With respect to cortical regions, an increase in OPC (nuclear OLIG1) density was detected in white matter adjacent to the BA32 and BA9 in MDD postmortem as compared to controls, while the density of mature oligodendrocyte (cytoplasmic OLIG1) or MBP level was unchanged, suggesting that changes in OPCs did not directly impact myelin content through the canonical myelinogenic pathway^39^. In contrast, an investigation through OPC-specific markers PDGFRA revealed a loss of OPCs in the frontal cortex of MDD postmortem samples compared to age-matched controls^51^. Based on complementary preclinical data, the authors proposed that reduction of OPCs would also imply decreased secretion of fibroblast growth factors 2 (FGF2) from OPCs. The latter would eventually impair astrocyte and neuron functions and the overall brain homeostasis^51^. Whilst the proposed working model is for sure intriguing, caution should be taken in drawing definitive conclusions, especially from the human dataset employed in this study. Specifically, major concerns emerge when statistically comparing the PMI of MDD and controls. Indeed, PMI was almost twice as high in MDD samples as compared to controls (MDD = 23 ± 10; Controls = 12 ± 6; mean ± SD), thus it cannot be excluded that the observed loss of OPCs was influenced by this technical discrepancy^51^.

Based on the well-established impact of aversive experiences during sensitive developmental windows^89^, three additional studies evaluated the effect of childhood trauma on the pathophysiology of MDD. Specifically, they compared postmortem control samples with postmortem samples from MDD individuals and MDD individuals who experienced child abuse (MDD-CA)^53,56,60^. Collectively, these studies confirmed that the OPC density was unchanged in BA9, BA11, BA12, BA24 and BA32 of MDD and MDD-CA compared with controls^53,56,60^, and identified brain region-specific changes in myelin content. Specifically, reduction of MBP, suggestive of myelin loss, was observed in the BA9 of both MDD and MDD-CA^53^, while reduced density of OLIG2^+^ cells and increased density of mature oligodendrocytes was exclusively observed in the BA9 of MDD-CA as compared to MDD and controls. Furthermore, reduction of SOX10^+^ cells and changes in myelin content were observed in the BA24 and BA32 white matter^60^. These findings suggest that alterations in the oligodendrocyte lineage maturation dynamics might be a distinctive pathophysiological mechanism specifically associated with early trauma-related MDD onset. Moreover, increased density of perineuronal nets (PNNs) and increased proportion of parvalbumin (PV) neurons surrounded by PNNs was observed in the BA-11-12 of MDD-CA, but not of MDD or controls^56^. Moreover, OPC-PV neuron proximity and PNN-related transcripts in OPCs were higher in MDD-CA as compared with MDD and controls and both parameters correlated with PNN density^56^.

Taken together, lines of evidence support the implication of OPCs alterations in the onset of MDD. Importantly, and to some extent in contrast with SCZ and BD, it appears that both the canonical and non-canonical OPCs pathways might be equally involved in the pathophysiology of MDD. Whether aversive experiences during a sensitive developmental window or differences in the disease symptomatology or brain region-related discrepancies might be the determinant factors for associating one or the other OPC pathways to the occurrence of MDD remains an open question.

##### Other mental disorders

Three additional research articles retrieved through our search strings dealt with psychiatric illnesses other than SCZ, BD, and MDD^61^ or combined multiple disorders and analysed them according to non-conventional diagnostic categories^62,63^. One study focused on post-traumatic stress disorder (PTSD), which is a trauma-related disorder that develops after experiencing at least one severe traumatic event/stressor^90^. Symptoms of PTSD can appear immediately after the traumatic experience or suddenly emerge after many years from the event; they include hyper-alertness, difficulties in concentrating and/or sleeping, guilt over the whole traumatic experience, emotional numbing, and re-experiencing/recalling (in dreams or thoughts) the traumatic events^72,90^. In this study, already available GWAS datasets were combined with data from proteome-wide association studies (PWASs), and from transcriptome studies (i.e. microarray and single-cell RNAseq data) to determine how certain risk loci for PTSD could impact downstream mRNA and protein levels and increase the likelihood of developing PTSD^61^. The authors identified seven risk genes – highly expressed in brain regions relevant to PTSD, such as hippocampus, amygdala, cingulate cortex and nucleus accumbens. Importantly, three of these most replicated genes, namely Ras-Related Protein Rab-27B (*RAB27b*), Leiomodin 1 (*LMOD1*) and Exocyst Complex Component 6 (*EXOC6*), were enriched in OPCs and excitatory neurons. Of these 3 most replicated candidates, *EXOC6* was included with high confidence. Given the involvement of EXOC6 in vesicular trafficking, it is intriguing to speculate that these findings might be suggestive of deficits in vesicle-mediated excitatory neuron-OPCs communication in the onset of PTSD^61^. Two additional studies focused on overall major mental illnesses. One of them specifically addressed the effects of *t*(1:11) translocation, known to be associated with the onset of various mental diseases^62^. Specifically, through iPSC technology combined with transplantation, it was shown that, in some instances, *t*(1:11) translocation led to the reduction of proliferative OPCs and to the increase of O4^+^ oligodendrocyte density, which were paralleled by additional changes in myelin- and oligodendrocyte-related transcripts and myelin loss^62^. The second study implemented a non-conventional classification for psychiatric pathologies and compared internalising disorders, externalising disorders, thought disorders with healthy controls^63^. Through a longitudinal approach, the authors observed that internalizing (i.e. depression, anxiety and fear) and externalising disorders (i.e. inattention, aggressive and disruptive behaviour), but not thought disorders (delusion, hallucination and obsession), associate with increased cortical thickness. Interestingly, volumetric changes in the left caudal middle frontal gyrus of patients affected by internalising disorders specifically associated with OPC- and GABAergic neuron-related common genetic variations, again pointing at the potential impairment of non-canonical OPC pathways, including neuron-OPC communication^63^. Taken together, these additional findings call attention on the necessity of further studies on OPC pathology and its implication in a variety of mental illnesses.

## Is there a future for oligodendrocyte precursor cells in psychiatry?

This review assembled the available evidence to date on the implication of OPC (dys)function in the onset (or aggravation) of various psychiatric illnesses, with a specific focus on clinical and post-mortem findings. To ensure a comprehensive and fully unbiased inclusion of the relevant research articles, we designed wide-ranging search strings for two databases. Despite the permissiveness of our searches and the substantial number of retrieved articles, only a relatively small proportion of these articles met the pre-defined inclusion criteria.

**Fig. 4:**
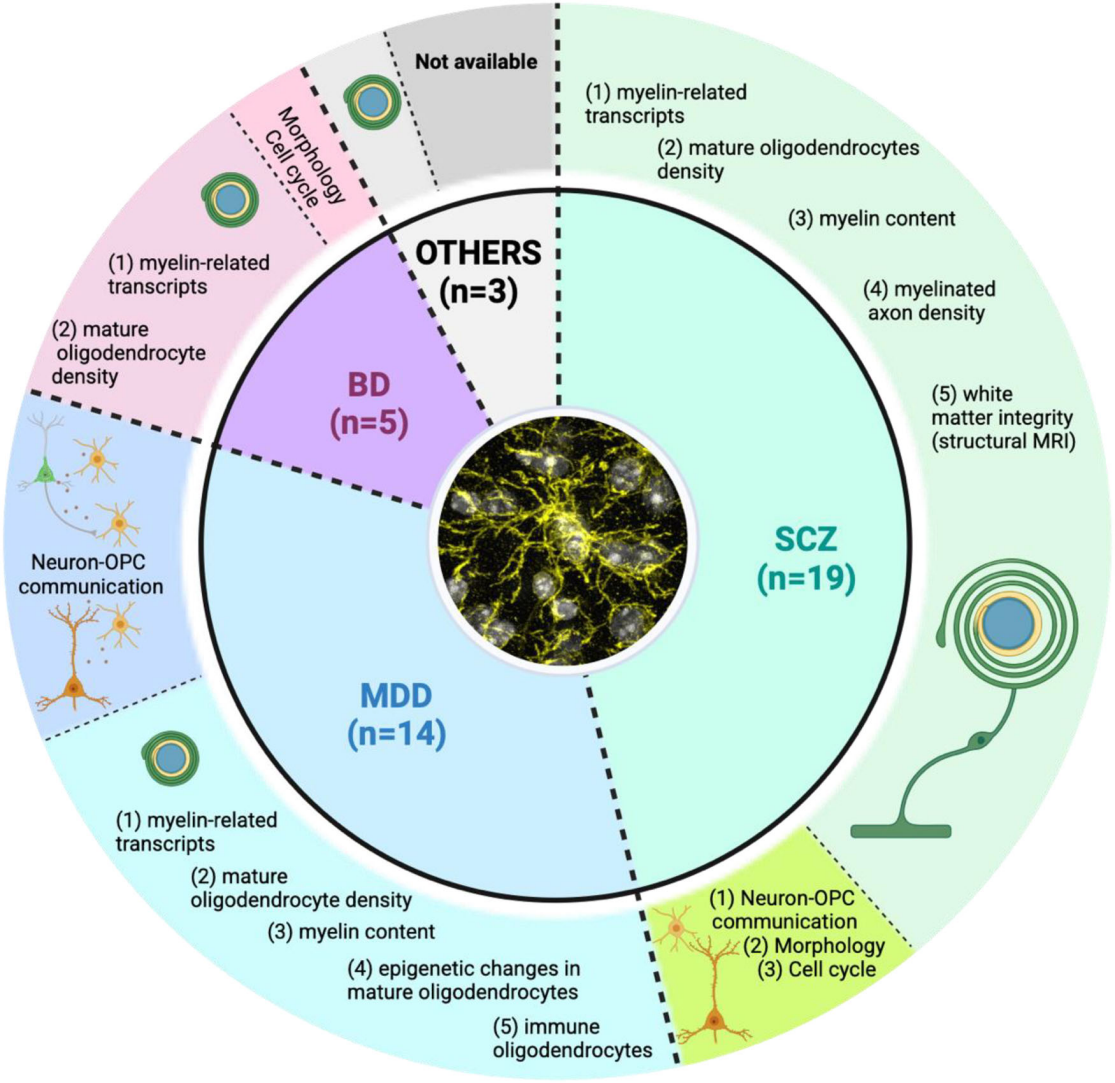
Graphical representation of the main findings in the oligodendrocyte lineage and mental illnesses. The internal pie-chart shows the proportion and the number of studies that investigated the included mental diseases (SCZ = schizophrenia; MDD = major depressive disorder; BD = bipolar disorder; Other = PTSD + any mental disease other than SCZ, MDD, BD). Diseases are separated by colour and through thick black dashed lines. The outer pie-chart show the proportion of studies, within the specific pathology, that reported on either canonical/myelinogenic or non-canonical OPC functions. Canonical and non-canonical pathways are separated by colour and thin black dashed lines. This figure has been created with Biorender (https://www.biorender.com).

From the biometric analysis, it emerged that the field of OPCs in psychiatry is at very early stage, with the first publication appearing only in 2003 and with most of the findings restricted to two psychiatric disorders, namely SCZ and MDD (Fig. 4). This for sure limits the understanding of the role of OPCs in the broad realm of mental illnesses. One explanation for such a narrow disease focus might be found in the limited availability of postmortem samples from other mental disorders, despite their high lifetime prevalence (e.g. anxiety disorders). Nevertheless, the existing data seem to support the involvement of OPCs in the occurrence of mental disorders, although the precise underlying pathological mechanisms remain elusive and the magnitude and the pattern of the contribution of OPC (dys)function to mental illnesses is still highly debatable. The latter is especially evident when screening comprehensive and unbiased cell type studies performed in post-mortem brains from psychiatric patients^59,91–118^. Indeed, through our *control search strategy*, we screened for studies that employed single-cell or single-nucleus RNA-sequencing in post-mortem brains of psychiatric patients and retrieved 27 articles that met the required inclusion criteria in such samples^59,91–113,115^. Of the latter, only 7 studies highlighted OPCs (MDD, *n* = 5; BD, *n* = 2; Anxiety disorder, *n* = 1)^54,57,59,97,100,101,108^. Also, it is important to point out that most of the MDD-related studies relied (partially or completely) on the same publicly available dataset from Nagy et al. ^54^. This might point out that OPCs are not necessarily the most prominent cell type in the pathophysiology of mental illness but might be involved in the occurrence of certain symptoms, be exclusively relevant for a certain subset of patients, and/or indirectly exert their effects through other glial cells or neurons.

When focusing on SCZ studies, it appears that most of the OPC-related alterations—when detected—ultimately impact on myelin content, although a few very recent findings also hint at dysfunction of the non-canonical OPC pathways, including alteration in neuron-OPC communication and morphological anomalies^48,49^. The limited findings on BD mostly overlap with what is observed in SCZ patients, suggesting that OPC alterations might be responsible for symptoms that are shared between these disorders, such as psychotic symptoms. This further supports the hypothesis that OPC (dys)function might be prominently related to certain symptoms shared between mental illnesses more than to a single mental disorder. In MDD the picture is somewhat more ambiguous. Indeed, whilst a substantial portion of the published articles reported on OPC alterations that were paralleled by myelination defects, a considerable number of studies also detected changes in OPCs that affected their non-canonical functions, including neuron-OPC communication^54^, brain homeostasis^51^ and formation/maintenance of perineural network around parvalbumin interneurons^56^. Whilst specific mechanisms underlining OPC dysfunctions in MDD might partially diverge from what is observed in SCZ, their timing might be one common denominator. Indeed, the early onset of SCZ and BD and the effects of early life (aversive) environmental stimuli on the onset of MDD emphasises the relevance of OPCs (dys)function during critical/sensitive developmental windows. Considering the emerging fundamental role of OPCs in brain circuit maturation^119,120^, early life deficits in OPCs could impair overall brain maturation and hinder cognitive and emotional functions later in life. Critically, this might represent a biological substrate—or aggravating phenomenon—shared by various psychiatric pathologies. In line with this “developmental hypothesis”, we propose that a special attention should be given to another cell population implicated in various psychiatric disorders, i.e. PV-expressing interneurons. This is especially relevant when also considering the shared origin with OPCs during early brain development^121,122^, which might be also critical for the understanding of disease mechanisms.

Importantly, as well as for MDD, PV+ interneurons have been implicated in the pathophysiology of SCZ. While the number or density of parvalbumin-positive interneurons seems to be unaffected, the mRNA expression levels were decreased in schizophrenia patient samples compared to the controls^123,124^, possibly hinting at alterations in the plasticity (and activity) of PV-interneurons^125–127^. To put this into context with oligodendrocyte lineage cells, altered myelination of specifically parvalbumin-positive interneurons was hypothesized to play a role in the pathogenesis of SCZ. These interneurons have been identified to be the predominantly myelinated interneurons in the cerebral cortex which is crucial for the proper synchronization of pyramidal neurons^128^. Whether early dysfunction in PV-OPCs mutual influence might initiate a pathological cascade that would lead to the occurrence of different psychiatric pathologies – as well as the related specific mechanism(s) – remain unexplored.

This review clearly exposes the heterogeneity of the findings in the field. Part of such heterogeneity might be purely methodological (e.g. differences in data analysis approaches) or be related to additional confounding variables, such as medications, age at death, cause of death and brain region under examination. However, the complexity and multifactorial nature of the pathologies under examination is probably the main cause of the apparent lack of coherence amongst findings. Future studies, possibly combining the investigation of independent human cohorts with the application of new technologies (e.g. multiomics approaches and multimodal magnetic resonance imaging techniques) and with the employment of valid preclinical models, will fill the current knowledge gaps and will consistently determine the extent by which OPCs contribute to the pathophysiology of mental diseases. Importantly, considering the complexity of mental disorders and the overlap of multiple symptoms and, relevant to the present context, the similarity of certain OPC-related alterations, two main approaches should be employed. The first approach should consider addressing how gene-environment interaction could lead to OPC alterations and, consequently, to the onset (or worsening) of mental illnesses. In fact, multiple lines of evidence point out how mental disorders might stem from the interplay between an unfavourable genetic constellation and adverse environmental stimuli (see eg^129,130^.). With respect to the second approach, it would be advantageous to adopt research frameworks, such as the Research Domain Criteria established by the National Institute of Mental Health^131^, and investigate mental health and illness in terms of changes of psychological and biological systems, rather than employing conventional disease classifications. Understanding these pathways and their dysfunctions in relation to specific pathological sub-phenotypes and/or symptoms may offer new avenues for patient-tailored therapeutic interventions in mental health conditions and, possibly, for preventive measures that could take advantage of OPCs plasticity and promote mental health rather than curing mental illnesses.

## Supplementary information

Supplementary Information

## Data Availability

No datasets were generated or analysed during the current study.
