# Supplementary material for: Canonical and non-canonical roles of oligodendrocyte precursor cells in mental disorders
Source: Npj Ment Health Res. 2025 May 15;4:19. doi: 10.1038/s44184-025-00133-x (PMC12081632; doi:10.1038/s44184-025-00133-x)
Supplement: Supplementary file 1 — Supplementary Information [file 44184_2025_133_MOESM1_ESM.pdf]

Supplementary information

Supplementary Note 1. Search strategy

To ensure a comprehensive selection of relevant articles, two databases were employed, i.e. PubMed (https://pubmed.ncbi.nlm.nih.gov/) and Web of Science (https://www.webofknowledge.com). The following search strings were applied, respectively:

Pubmed.

((psychiatric illnesses) OR (psychiatry) OR (neuropsychiatric diseases) OR (affective disorder) OR (Depression) OR (Anxiety) OR (schizophrenia) OR (bipolar) OR (trauma) OR (PTSD) OR (addiction)) AND((OPCs) OR (oligodendrocyte precursor cells) OR (oligodendrocyte progenitor cells) OR (oligodendrocyte progenitor) OR (NG2 glia))

Web of Science.

(AB=(psychiatric illnesses OR psychiatry OR neuropsychiatric diseases OR affective disorder OR depression OR anxiety OR schizophrenia OR bipolar OR trauma OR PTSD OR addiction)) AND (AB=(OPCs OR oligodendrocyte precursor cells OR oligodendrocyte progenitor cells OR oligodendrocyte progenitor OR NG2 glia)) OR (TI=(psychiatric illnesses OR psychiatry OR neuropsychiatric diseases OR affective disorder OR depression OR anxiety OR schizophrenia OR bipolar OR trauma OR PTSD OR addiction)) AND (TI=(OPCs OR oligodendrocyte precursor cells OR oligodendrocyte progenitor cells OR oligodendrocyte progenitor OR NG2 glia))

In the search process, no time and language limit was set so that all possible relevant studies be retrieved by May 18, 2024. A total of n=1451 articles were retrieved (Pubmed, n=1170; Web of Science, n=281).

Inclusion criteria

1. Original research papers
2. English
3. Available full test
4. Any study design that allows to determine the relevance of OPCs in psychiatry
5. Human pathologies: schizophrenia (SCZ), bipolar disorder (BD), anxiety disorders (ADs), post-traumatic stress disorder (PTSD), traumatic experience/severe stress, major depressive disorder (MDD), and psychiatric comorbidities
6. Human studies

Exclusion criteria

1. Any format other than research paper, e.g. review article, commentary, perspective.
2. Any language other than English
3. Unavailability of the online version of the full text
4. Studies not addressing OPCs in psychiatry and/or preclinical models of psychiatric symptoms
5. Human studies addressing organic and neurological comorbidities with the psychiatric pathologies
6. Preclinical models exploring symptoms clearly relevant to psychiatric illnesses, but also to other non-psychiatric diseases (no comorbidity).
7. Preclinical models
8. In vitro studies

Supplementary Note 2. Control search strategy

Pubmed.
((psychiatric illnesses) OR (psychiatry) OR (neuropsychiatric diseases) OR (affective disorder) OR (Depression) OR (Anxiety) OR (schizophrenia) OR (bipolar) OR (trauma) OR (PTSD) OR (addiction)) AND ((scRNA seq) OR (single-cell RNA sequencing))

Web of Science.
(AB=(psychiatric illnesses OR psychiatry OR neuropsychiatric diseases OR affective disorder OR depression OR anxiety OR schizophrenia OR bipolar OR trauma OR PTSD OR addiction)) AND (AB=(single-cell RNA sequencing OR scRNA seq)) OR (TI=(psychiatric illnesses OR psychiatry OR neuropsychiatric diseases OR affective disorder OR depression OR anxiety OR schizophrenia OR bipolar OR trauma OR PTSD OR addiction)) AND (TI=(single-cell RNA sequencing OR scRNA seq))

In the search process, no time and language limit was set so that all possible relevant studies be retrieved by March 17, 2025. A total of n=1442 articles were retrieved (Pubmed, n=1220; Web of Science, n=222).

Inclusion criteria

1. Original research papers
2. English
3. Available full test
4. Any study design that allows to highlight cell populations relevant to psychiatric illnesses
5. Human pathologies: schizophrenia (SCZ), bipolar disorder (BD), anxiety disorders (ADs), post-traumatic stress disorder (PTSD), traumatic experience/severe stress, major depressive disorder (MDD), and psychiatric comorbidities
6. Human studies

Exclusion criteria

1. Any format other than research paper, e.g. review article, commentary, perspective.
2. Any language other than English
3. Unavailability of the online version of the full text
4. Human studies addressing organic and neurological comorbidities with the psychiatric pathologies
5. Preclinical models exploring symptoms clearly relevant to psychiatric illnesses, but also to other non-psychiatric diseases (no comorbidity).
6. Preclinical models
7. In vitro studies

Supplementary table 1. Summary of the included studies: demographic and methods.

Publication	Pathology	Age	Sex	Sample size	Sample type | *Source*	Method(s)	OPC Marker(s)
Tkachev et al 2003	BD	BD = 42.3 (25–61)<br>CON = 48.1 (29–68)	M/F	BD, n=15<br>CON, n=15	Brodmann area 9<br>*Stanley brain collection*	Affymetrix Genome U133A array<br>qPCR	*PDGFRA*<br>*NG2*<br>*OLIG1*<br>*OLIG2*<br>*SOX10*
Tkachev et al 2003	SCZ	SCZ = 44.2 (25–62)<br>CON = 48.1 (29–68)	M/F	SCZ, n = 15<br>CON, n=15	Brodmann area 9<br>*Stanley brain collection*	Affymetrix Genome U133A array<br>qPCR	*PDGFRA*<br>*NG2*<br>*OLIG1*<br>*OLIG2*<br>*SOX10*
Aston et al 2005	MDD	MDD = 46±3<br>CON = 49±3<br>(mean±SEM)	M/F	MDD<br>First round, n=12<br>Follow-up, medication-free, n=5;<br>suicide-free, n=7<br><br>CON, n=14	Temporal cortex (middle temporal gyrus)<br>*The Stanley Consortium*	Affymetrix HgU95A microarrays<br>(12'000 genes)	*SOX10*
Iwamoto et al 2005	SCZ	SCZ = 44.2 (25–62)<br>CON = 48.1 (29–68)	M/F	SCZ, n = 11<br>CON, n = 12	Prefrontal cortex (BA10)<br>*Stanley Foundation*	Bisulfite sequencing	*SOX10*
Katsel et al 2005	SCZ	SCZ = 74.7 ± 2.37<br>CON = 80.7 ± 3.02	M/F	SCZ, n = 9-21<br>CON, n = 8-18<br>(Number of subjects varied depending on the brain ragion)	Multiple brain regions<br>*Brain Bank of the Department of Psychiatry of the Mount Sinai Medical Center (New York, NY)/Veter- ans Administration Medical Center (Bronx, NY).*	HG-U133 A and B Human genome Gen-eChipR	*OLIG2*<br>*SOX10*
Mitkus et al 2007	SCZ	**White matter**<br>SCZ = 48.8 ± 15.9<br>CON = 41.4 ± 15.1<br><br>**Grey Matter**<br>SCZ = 46.8 ± 15.3<br>CON = 41.2 ± 14.7	M/F	**White matter**<br>SCZ, n = 33<br>CON, n =73<br><br>**Grey Matter**<br>SCZ, n = 31<br>CON, n =68	Dorsolateral prefrontal cortex<br>*Clinical Brain Disorders Branch, NIMH*	qPCR	*OLIG2*

Saetre et al 2007	SCZ	SCZ = 54.7 ±17.3<br>CON = 58.5 ± 18.0<br>(mean±SD)	M/F	SCZ, n=55<br>TYP, n=19<br>ATYP, n=7;<br>NTD, n=11<br>CON, n=55	Brodmann area 8 and 9 and the left side of the superior frontal gyrus<br>*Harvard and Stanley brain banks & Maudsley brain bank*	Global and high-resolution mRNA quantification	*OLIG2*
Barley et al 2009	SCZ	SCZ = 45.8 (3.7) | 45.8 (3.7) | 43.6 (3.5) | 43.6 (3.5)<br>CON = 48.1 (2.8) | 48.1 (2.8) |48.1 (2.8) |49 (2.8)	M/F	SCZ, n=12-14<br>CON, n =14-15	Multiple brain regions<br>*Stanley Foundation Neuropathology Consortium (SFNC).*	qPCR	*NGN3*<br>*NG2*<br>*PDGFRα*<br>*SOX4*<br>*SOX11*<br>*SOX10*<br>*OLIG2*
Barley et al 2009	BD	BD = 45.9 (2.5) |45.8 (2.7) | 46.1 (2.5) |46.8 (3.1)<br>CON = 48.1 (2.8) | 48.1 (2.8) |48.1 (2.8) |49 (2.8)	M/F	BD, n= 11-14<br>CON, n =14-15	Multiple brain regions<br>*Stanley Foundation Neuropathology Consortium (SFNC).*	qPCR	*NGN3*<br>*NG2*<br>*PDGFRα*<br>*SOX4*<br>*SOX11*<br>*SOX10*<br>*OLIG2*
Barley et al 2009	MDD	MDD = 42.9 (3.7) | 41.9 (3.2) | 41.9 (3.2) | 41.9 (3.5)<br>CON = 48.1 (2.8) | 48.1 (2.8) |48.1 (2.8) |49 (2.8)	M/F	MDD, n= 11 -14<br>CON, n =14-15	Multiple brain regions<br>*Stanley Foundation Neuropathology Consortium (SFNC).*	qPCR	*NGN3*<br>*NG2*<br>*PDGFRα*<br>*SOX4*<br>*SOX11*<br>*SOX10*<br>*OLIG2*
Kerns et al 2010	SCZ	SCZ = 43.6 ±3.5<br>CON = 48.1 ± 2.8<br>(48.5 ±2.9)<br>(mean±SEM)	M/F	SCZ, n=14<br>CON, n=14-15	Posterior limb of the internal capsule (ICp)<br>*Stanley Foundation Neuropathology Consortium*	Real Time qPCR<br>Histology	***Real Time qPCR***<br>Cell cycle genes: *CCND1*, *CCND2*, $p21^{Cip1}$, $p27^{Kip1}$, $p57^{Kip2}$<br>Notch Pathway: *NOTCH1*, *JAG1*, *HES1*, *HES5*, and *DTX1*<br>Apoptosis: *CASP3*<br>Cellular Proliferation: *PCNA*<br><br>***Histology***<br>Nissl staining
Kolomeets et al 2013	SCZ	SCZ = 39.8 ± 10.7<br>CON = 44.3 ± 9.3<br>(mean±SD?)	M/F	SCZ, n = 24<br>CON, n= 24	Brodmann area 39 and 40<br>*Stanley "Parietal Collection"*	Histology	Numerical density of oligodendrocyte clusters (Nissl staining)

Mosebach et al 2013	MDD	MDD = 47±14<br>CON = 55±12<br>(mean±SD)	M/F	MDD, n=9<br>CON, n= 16	Pregenual anterior cingulate (pACC)/dorsolateral prefrontal cortex (DLPFC), and adjacent white matter<br>*Magdeburg brain bank*	Histology	OLIG1 (nuclear)
Mosebach et al 2013	BD	BP = 56±11<br>CON = 55±12<br>(mean±SD)	M/F	BP, n=8<br>CON, n= 16	Pregenual anterior cingulate (pACC)/dorsolateral prefrontal cortex (DLPFC), and adjacent white matter<br>*Magdeburg brain bank*	Histology	OLIG1 (nuclear)
Mosebach et al 2013	SCZ	SCZ = 54±9<br>CON = 55±12<br>(mean±SD)	M/F	SCZ, n= 13<br>CON, n= 16	Pregenual anterior cingulate (pACC, Brodmann Area 32) Dorsolateral prefrontal (DLPFC, Brodmann Area 9) Adjacent white matter<br>*Magdeburg brain bank*	Histology	OLIG1 (nuclear)
Birey et al 2015	MDD	MDD = 39.08 ± 17.35<br>CON = 53.75 ± 21.95<br>(mean±SD)*	M/F	MDD, n=12<br>CON, n=8	Frontal cortex<br>*Nichd brain and tissue bank for developmental disorders (Maryland)*	Western Blot<br>Histology	PDGFRA<br>NG2
Manuey et al 2015	SCZ	SCZ = 64.8 ± 7.6<br>CON = 65.1 ± 8.4	M/F	SCZ, n=9<br>CON, n= 9	Brodmann area 9<br>*Harvard Brain Tissue Resource Center*	Immunohistochemistry	NG2<br>OLIG2
Rajkowska et al 2015	MDD	MDD = 55 ± 4 (20-87)<br>CON = 52 ± 4 (27-80)	M/F	MDD, n=20<br>CON, n=16	Prefrontal cortex<br>*Cuyahoga County Medical Examiner's Office (Cleveland, OH)*	qPCR	OLIG1
Saia-Cereda et al 2016	SCZ	SCZ = 66 ± 14<br>CON = 59 ± 14<br>(mean±SD)*	M/F	SCZ, n=5<br>CON, n= 5	Corpus callosum<br>*Psychiatric Center Nordbaden, Wiesloch, Germany (patient) & Institute of Neuropathology, Heidelberg University, Heidelberg, Germany (control)*	Phosphoproteomic (shotgun mass spectrometry)	Ephrin B signaling pathway:<br>GNB4 (Guanine nucleotide binding protein)<br>VAV2 (guanine nucleotide exchange factory)

Lutz et al 2017	MDD | Suicide | Child abuse	MDD-CA = 41.6 ± 2.8<br>MDD = 48.2 ± 2.3<br>CON = 46.3 ± 4.1<br>(mean±SEM)	M/F	MDD-CA = 27<br>MDD = 25<br>CON = 26	Anterior cingulate cortex (Brodmann 24 and Brodmann 32)<br>*Douglas-Bell Canada Brain Bank*	Genome.wide DNA methylation<br>Histology / Stereology<br>Bulk RNA-seq	PDGFRA<br>SOX-10
Windrem et al 2017	SCZ	SCZ = 13 ± 2<br>CON = 27 ± 4<br>(mean±SD)*	M/F	SCZ, n = 5 (7 cell lines)<br>CON, n = 3 (3 +1 cell lines)	Glia progenitor cells (hGPCs) derived from SCZ and CON and transplanted in *Shiverer* mice	RNAseq<br>qPCR<br>Western blot<br>Histology	Multiple OPCs-related transcripts
McPhie et al 2018	SCZ	**Skin biopsy**<br>SCZ = 39.7 ± 4.8<br>CON = 29.7 ± 9.9<br><br>**MR scan**<br>SCZ = 40.0 ± 6.1<br>CON = 30.5 ± 10.3<br>(mean±SD)	M/F	SCZ (or schizoaffective disorders), n= 6<br>CON, n= 6	- Human OL differentiated from IPSCs<br>- White matter of the right prefrontal cortex (MTR)<br>- Whole brain segmentation (T1-weighted)	Single Cell RNAseq<br>Immunofluorescence<br>Magnetization Transfer Ratio (MTR) and T1-weighted<br>Exome Sequencing	OLIG2<br>SOX10<br>$O4^+$<br>CSPG4 (exon rare variants)
Tanti et al 2018	MDD | Suicide | Child abuse	MDD-Suicide-CA = 37.2 ± 11.0<br>MDD-Suicide = 45.5 ± 12.7<br>CON = 37.9 ± 13.7<br>(mean±SEM)	M	MDD-Suicide-CA, n =18<br>MDD-Suicide, n= 18<br>CON, n=18	Ventromedial prefrontal white matter (adjacent BA11, BA12 and BA32)<br>*Suicide section of the Douglas-Bell Canada Brain Bank (Douglas Institute, Montreal, Canada)*	Histology<br>Immunoblotting	PDGFRA<br>OLIG2<br>$OLIG2^+/CC1^-$<br>$SOX10^+/NOGOA^-$

de Vrij et al 2019	SCZ	NA	M (discovery family)	*Discovery Sample*<br>SCZ, n = 5<br><br>*Sanger Sequencing - Independent cohort*<br>SCZ, n = 1219<br>General Population, n = 10611<br><br>*iPSCs cells*<br>SCZ (CSPG44A131T), n=3<br>CON, n=3<br><br>*MRI - DTI*<br>SCZ (CSPG44A131T), n= ?<br>Healthy sibilings non-carrier, n= ?<br>General population, n=294	Peripheral Blood DNA<br>Human iPSCs-derived OPCs<br>Whole brain (DTI)<br><br>*Non-consanguineous family of Dutch ancestry*<br>*Independent Dutch SCZ & Rotterdam Study cohort*	Illumina HumanCytoSNP-12v2 chip arrays<br>(Linkage and copy number analysis)<br>TaqMan Genotyping & Sanger Sequencing<br>Myelination assay on oeganitypic *Shiverer* slices<br>Magnetic Resonance Imaging (MRI) - DTI	CSPG4 ($CSPG4^{A131T}$ and $CSPG4^{V901G}$)
Papiol et al 2019	SCZ	SCZ - aerobic intervention = 37.3±11.7<br>SCZ - table soccer = 35.8±14.4<br>CON - aerobic intervention = 37.3±11.1<br>(mean±SD)	M/F	SCZ - aerobic intervention, n=20<br>SCZ - table soccer, n=21<br>CON, n=23	Hippocampus (in vivo MRI)<br>DNA from blood<br><br>*Department of Psychiatry and Psychotherapy of the University Medical Center Goettingen*	SNPs Genotyping<br><br>MRI T1-weighted magnetization- prepared rapid gradient echo (MP-RAGE)	5% most specific transcript to OPCs from Skene et al 2018 (including NG2 and PDGFRa)
Vasistha et al 2019	Major Mental Illnesses (MMIs)	NA	M (MMI) | M/F (CON)	*Whole-brain MRI*<br>MMI, n=8<br>CON, n=13<br><br>*iPSCs-derived OPCs*<br>MMI, n=4<br>CON, n=3	iPSC-derived OPCs<br>Whole-brain MRI<br><br>*Reported extended Scottish family*	iPSC-derived OPCs<br>qRT-PCR<br>RNAseq<br>diffusion MRI (dMRI), T1-weighted	PDGFRA<br>PDGFRA/EdU
Kolomeetss et al 220	MDD	MDD = 46.5 ± 9.3<br>CON = 48.1 ± 10.7<br>(mean±SD)	M/F	MDD, n=15<br>CON, n=15	Putamen<br>*SMRI Neuropathology Con- sortium Collection*	Histology	**Numerical density of oligodendrocyte clusters**<br>(Nissl staining)

Kolomeetss et al 220	BD	BD = 42.3 ± 11.7<br>CON = 48.1 ± 10.7<br>(mean±SD)	M/F	BD, n=15<br>CON, n=15	Putamen<br>*SMRI Neuropathology Con- sortium Collection*	Histology	**Numerical density of oligodendrocyte clusters**<br>(Nissl staining)
Kolomeetss et al 220	SCZ	SCZ = 44.5 ± 13.1<br>CON = 48.1 ± 10.7<br>(mean±SD)	M/F	SCZ, n=15<br>CON, n=15	Putamen<br>*SMRI Neuropathology Con- sortium Collection*	Histology	**Numerical density of oligodendrocyte clusters**<br>(Nissl staining)
Nagy et al 2020	MDD | Suicide	MDD = 41.06 ± 4.66<br>CON = 38.71 ± 4.32<br>(mean±SEM)	M	MDD, n = 17 (5 for RNAscope)<br>CON, n = 17 (5 for RNAscope)	Brodmann area 9<br>*Douglas–Bell Canada Brain Bank*	Single-nucleus transcriptomics<br>RNAscope<br>High-throughput PCR	Multiple OPC-specific transcripts
Di Biase et al 2022	SCZ	**Discovery Cohort**<br>SCZ = 22.782 ± 3.83<br>CON = 24.30 ±4.10<br><br>**Validation Cohort**<br>SCZ = 39.70 ± 10.81<br>CON = 41.05 ± 14.02<br>(mean±SD)	M/F	**Discovery cohort**<br>SCZ, n = 140<br>CON, n = 1267<br><br>**Validation cohort**<br>SCZ, n=335<br>CON, n=185<br><br>**Gene expression map reference**<br>CON, n=6	34 cortical regions (MR)<br>DNA (SNPs genotyping)<br><br>*(1) Human Connectome Project-Young Adult Sample*<br>*(2) Human Connectome Project for Early Psychosis*<br>*(3) Australian Schizophrenia Research Bank*<br><br>*Allen Brain Institute*	T1-weighted MRI<br>SNPs Genotyping<br>Gene-expression	OPC-related genetic load
Kokkosis et al 2022	MDD | Suicide	MDD = 41.06 ± 4.66<br>CON = 38.71 ± 4.32<br>(mean±SEM)<br>*(Nagy et al 2020)*	M	MDD, n = 17<br>CON, n = 17	Brodmann area 9<br>*Douglas–Bell Canada Brain Bank*	Single-nucleus transcriptomics (usage of already published data)	Multiple OPC-related transcripts, including *PDGFRA*, *CSPG4*, *OLIG2*, *OLIG1*.<br>Cluster: OPCs, Committed-OPC

Tanti et al 2022	MDD | Child abuse	MDD-CA = 37.75 ± 3.10<br>MDD = 46.63 ± 3.48<br>CON = 43.18 ± 7.11<br>(mean±SEM)"<br><br>**Single-nucleus transcriptomics**<br>MDD = 41.06 ± 4.66<br>CON = 38.71 ± 4.32<br>(mean±SEM)<br>(Nagy et al 2020)	M/F	MDD-CA, n = 12<br>MDD, n = 16<br>CON, n = 11<br><br>**Single-nucleus transcriptomics**<br>MDD, n = 17<br>CON, n = 17 "	Brodmann area 11/12<br>*Douglas–Bell Canada Brain Bank*<br><br>Brodmann area 9<br>*Douglas–Bell Canada Brain Bank*	Immunostaining<br>In situ hybridization<br>Single-nucleus transcriptomics	PDGFRA
Wingo et al 2022	PTSD	Adulthood	NA	**Proteomics**<br>n=525 brains<br><br>**GWAS**<br>n=186689 participants<br><br>**Cell type-specific profiling**<br>n=24 cognitively normal donors (single-cell RNAseq data)<br>n=6 neurotypical adults (RNA microarray data)	Dorsolateral prefrontal cortex (proteomics)<br>dPFC, frontal cortex, temporal cortex, inferior frontal gyrus, superior temporal gyrus, perirhinal gyrus.<br>*European descent of the Religious Orders Study and Rush memory project*<br>*Banner Sun Health Research Insitute*<br>*Mount Sinai Brain Bank*	Transcriptome-wide association study (TWAS)<br>Proteom-wide association study (PWAS)	RAB27b<br>EXOC6<br>LMOD1
Yu et al 2022	SCZ	SCZ = 57 ± 18<br>CON = 56 ± 15<br>(mean±SD)*	M/F	SCZ, n = 5<br>CON, n = 5	Cortex, hippocampus, amygdala<br>*National Health and Disease Human Brain Tissue Resource Center at Zhejiang University in China*	Immunohistochemistry	NG2<br>OLIG2
Maitra et al 2023	MDD	MDD - F = 45.10 ± 3.19<br>CON - F = 47.89 ± 4.45<br><br>MDD - M = 41.06 ± 4.66<br>CON - M = 38.38 ± 4.58	M vs F	MDD - F, n= 20<br>CON - F, n = 18<br><br>MDD - M, n = 17<br>CON - M, n = 16	Brodmann area 9<br>*Douglas–Bell Canada Brain Bank*	Single-nucleus transcriptomics	Multiple OPCs-specific transcripts

Yu et al 2023	Multiple disorders	Internalising disorders = 119.24 ± 7.54<br>Externalising disorders = 119.18 ± 7.39<br>Thought disorders = 118.79 ± 7.42<br>CON = 119 ± 7.48<br>(Age, mean ± SD, mo)	M/F	Internalising disorders, n = 1959<br>Externalising disorders, n = 1182<br>Thought disorders, n = 347<br>CON, n = 4041	Whole-brain CT<br>Cognitive screening<br>Samples for SNPs screening (e.g. blood, hair…)<br>*ABCD Study (Release 3.0, November 2020)*	MRI Scan<br>Psychological tests<br>Genome-wide association study	OPC-related genetic load
Zhou et al 2023	MDD | Suicide	**Combination of 4 datasets:**<br>GSE102556 (mRNA)<br>MDD-M = 46.7±15.7; CON-M=41.2±11.3<br>MDD-F = 43.7±11.6; CON-F=58.1±19.5<br><br>GSE88890 (methylation)<br>BA11: MDD = 48.6 ±20.8; CON = 39.4±19.5<br>BA25: MDD = 49.5 ±22.4; CON = 41.2±19.6<br>(mean±SD)<br><br>GSE144136 (single-nucleus RNAseq)<br>MDD = 41.06 ± 4.66; CON = 38.71 ± 4.32<br>(mean±SEM)<br><br>GSE197622 (single-nucleus RNAseq)<br>60-day old<br><br>**Post-morten BA11 and BA25:**<br>BA11: MDD = 45 ±15; CON = 51±20<br>BA25: MDD = 42 ±14; CON = 41±9<br>(mean±SD)	M/F	**Combination of 4 datasets:**<br>GSE102556 (mRNA)<br>MDD-sucide, n=37; CON, n=11;<br><br>GSE88890 (methylation)<br>MDD-sucide, n=20; CON, n=20;<br><br>GSE144136 (single-cell RNAseq)<br>MDD, n=17; CON, n=17<br><br>GSE197622 (single-nucleus RNAseq)<br>n=40 rats<br><br>**Post-morten BA11 and BA25:**<br>MDD, n= 5-6<br>CON, n= 3-4	Brodmann area 9<br><br>Brodmann area 11, Brodmann area 25<br>*Multiple Dataset & Douglas Bell Canada Brain Bank*	Differential Gene Expression (DGE)<br>Differential Methylated Regions (DMR)<br>Cross-omics correlation analysis<br>Functional annotation	Multiple OPCs-specific transcripts

Aranda et al 2024	SCZ	SCZ, n = 57.19 ±18.39<br>CON, n = 52.87±27.76<br>(mean ±SD)	M/F	SCZ, n = 563<br>CON, n = 963	Dorsolateral prefrontal cortex<br>*6 different collections of the PsychENCODE project: BrainGVEX, BrainSpan, CMC, BipSeq, LIBD and CMC-HBCC*	Bulk RNAseq<br>(usage of already published data)	Multiple OPC-specific transcripts
Aranda et al 2024	BD	BD, n = 45.48±13.91<br>CON, n = 52.87±27.76<br>(mean ±SD)	M/F	BP, n = 222<br>CON, n = 963	Dorsolateral prefrontal cortex<br>*6 different collections of the PsychENCODE project: BrainGVEX, BrainSpan, CMC, BipSeq, LIBD and CMC-HBCC*	Bulk RNAseq<br>(usage of already published data)	Multiple OPC-specific transcripts
Xie et al 2024	MDD	GSE144136 (single-nucleus RNAseq)<br>MDD = 41.06 ± 4.66;<br>CON = 38.71 ± 4.32<br>(mean±SEM)	M/F	GSE144136 (single-cell RNAseq)<br>MDD, n=17; CON, n=17	Brodmann area 9<br>*Douglas–Bell Canada Brain Bank*	Single-nucleus transcriptomics	Multiple OPC-specific transcripts

Supplementary table 2. Summary of the included studies: demographic, markers and main findings.

Publication	Pathology	Sample type | Source	Method(s)	Findings			
				OPCs	Myelin pathway	Other pathways	Non-conventional OPC pathways?
Tkachev et al 2003	BD	Brodmann area 9<br>*Stanley brain collection*	Affymetrix Genome U133A array<br>qPCR	=*PDGFRA*<br>= *NG2*<br>↓*OLIG1*<br>↓*OLIG2*<br>↓*SOX10*	Reduction of various myelin-related transcripts<br>↓*OLIG1*<br>↓*OLIG2*<br>↓*SOX10*	NA	NO
Tkachev et al 2003	SCZ	Brodmann area 9<br>*Stanley brain collection*	Affymetrix Genome U133A array<br>qPCR	= *PDGFRA*<br>= *NG2*<br>↓*OLIG1*<br>↓*OLIG2*<br>↓*SOX10*	Reduction of various myelin-related transcripts<br>↓*OLIG1*<br>↓*OLIG2*<br>↓*SOX10*	NA	NO
Aston et al 2005	MDD	Temporal cortex (middle temporal gyrus)<br>*The Stanley Consortium*	Affymetrix HgU95A microarrays<br>(12'000 genes)	⇣ *SOX10*	Reduction of myelin-related transcripts	- Axonla growth<br>- Synaptic functions<br>- Cell-cell communication	NO
Iwamoto et al 2005	SCZ	Prefrontal cortex (BA10)<br>*Stanley Foundation*	Bisulfite sequencing<br>qPCR	↓*SOX10*<br>↑Methylation of SOX10 CpG	Methylation of SOX10 impacts on various myelin-related gene expression	NA	NO
Katsel et al 2005	SCZ	Multiple brain regions<br>*Brain Bank of the Department of Psychiatry of the Mount Sinai Medical Center (New York, NY)/Veter- ans Administration Medical Center (Bronx, NY).*	HG-U133 A and B Human genome Gen- eChipR	⇣ *SOX10*<br>⇣*OLIG2*	Reduction of myelin-related transcripts	Implication of multiple pathways, including presynaptic secretory release, postsynaptic functions, energy metabolism	NO
Mitkus et al 2007	SCZ	Dorsolateral prefrontal cortex<br>*Clinical Brain Disorders Branch, NIMH*	qPCR	= *OLIG2*	Reduction of myelin-related transcripts and proteins	NA	NO

Saetre et al 2007	SCZ	Brodmann area 8 and 9 and the left side of the superior frontal gyrus *Harvard and Stanley brain banks & Maudsley brain bank*	Global and high-resolution mRNA quantification	↓*OLIG2* = mediated by antipsychotic treatment	Reduction of myelin-related transcripts (antipsychotic effect?)	Inflammatory pathway:<br>- alpha-1-antichymotrypsin (SERPINA3),<br>- interferon- induced transmembrane 2 and 3 (IFITM2 and IFITM3),<br>- guanylate binding protein 1 interferon inducible (GBP1)<br>- major histocompatibility complex, class1, A (HLA-A)	NO
Barley et al 2009	SCZ	Multiple brain regions *Stanley Foundation Neuropathology Consortium (SFNC).*	qPCR	↑*NG2* in putamen	Reduction in myelin-related transcripts	Increased in astrocyte-related transcripts	
Barley et al 2009	BD	Multiple brain regions *Stanley Foundation Neuropathology Consortium (SFNC).*	qPCR	↑*NG2* in putamen	No changes	No changes in astrocyte-related transcripts	
Barley et al 2009	MDD	Multiple brain regions *Stanley Foundation Neuropathology Consortium (SFNC).*	qPCR	No changes	↑*FYN* tyrosine kinase expression (mature oligodendrocyte)	Increased in astrocyte-related transcripts	
Kerns et al 2010	SCZ	Posterior limb of the internal capsule (ICp) *Stanley Foundation Neuropathology Consortium*	Real Time qPCR<br>Histology	↓ *CASP3*, *PCNA*<br>↑ *JAG1*, *CCND2*<br>↓ *p27Kip1*, *p57Kip2*<br>↑ *CCND1*, *p21Cip1*, *DTX1*<br>= *NOTCH1*, *HES5*, *HES1*<br>↓ Oligodendrocyte density<br><br>Correlation:<br>CCND2 vs. NG2 (after Bonferroni Correction)	↓ Oligodendrocyte density	Correlation:<br>*CASP3* vs *ALDH1L1* & *GFAP*<br>*CCND1* & *CCND2* vs. *ALDH1L1* & *GFAP*<br>(not after Bonferroni Correction)	NO

Kolomeets et al 2013	SCZ	Brodmann area 39 and 40<br>*Stanley "Parietal Collection"*	Histology	↓ oligodendrocyte clusters density in layer 3 of BA39 and BA40 for adolescent onset of the disease<br>Loss of interhemispheric asymmetry	Loss of correlation between oligodendrocyte cluster density and oligodendrocyte density	NA	NO
Mosebach et al 2013	MDD	Pregenual anterior cingulate (pACC)/dorsolateral prefrontal cortex (DLPFC), and adjacent white matter<br>*Magdeburg brain bank*	Histology	↑OLIG1 (nuclear)$^+$ cells in white matter adjacent to pACC<br>= OLIG1 (nuclear)$^+$ cell in other analysed regions	= oligodendrocyte density (Nissl staining)<br>= OLIG1 (cytoplasmic)$^+$ cell in regions other than white matter adjacent pACC<br>= MBP$^+$ fiber density	= GFAP$^+$ cells density	NO
Mosebach et al 2013	BD	Pregenual anterior cingulate (pACC)/dorsolateral prefrontal cortex (DLPFC), and adjacent white matter<br>*Magdeburg brain bank*	Histology	= OLIG1 (nuclear)$^+$ cells	= oligodendrocyte density (Nissl staining)<br>= OLIG1 (cytoplasmic)+ cell	= GFAP$^+$ cells density	NO
Mosebach et al 2013	SCZ	Pregenual anterior cingulate (pACC, Brodmann Area 32) Dorsolateral prefrontal (DLPFC, Brodmann Area 9) Adjacent white matter<br>*Magdeburg brain bank*	Histology	= OLIG1 (nuclear)$^+$ cells	= oligodendrocyte density (Nissl staining)<br>= OLIG1 (cytoplasmic)+ cell	= GFAP$^+$ cells density	NO
Birey et al 2015	MDD	Frontal cortex<br>*Nichd brain and tissue bank for developmental disorders (Maryland)*	Western Blot<br>Histology	↓PDGFRa protein level (% over CON)<br>↓NG2$^+$ cells (% over CON)	NA	In preclinical model (CSD): Secretion of FGF2 from NG2, which can impact on neuron and astrocyte functions	YES
Manuey et al 2015	SCZ	Brodmann area 9<br>*Harvard Brain Tissue Resource Center*	Immunohistochemistry	=NG2$^+$ cells density<br>↓OLIG2$^+$ cells density	Microarray of CNPase-immunoreactive cells: involvement of proliferation & differentiation pathways	NA	NO

Rajkowska et al 2015	MDD	Prefrontal cortex<br>*Cuyahoga County Medical Examiner's Office (Cleveland, OH)*	qPCR	↑*OLIG1*	Alterations in myelin-related transcripts<br>Reduction of CNPase protein, despite the increased mRNA<br>Smaller Oligodendrocyte size	NA	NO
Saia-Cereda et al 2016	SCZ	Corpus callosum<br>*Psychiatric Center Nordbaden, Wiesloch, Germany (patient) & Institute of Neuropathology, Heidelberg University, Heidelberg, Germany (control)*	Phosphoproteomic (shotgun mass spectrometry)	↑GNB4 phosphorylation<br>↑VAV2 phosphorylation	Not detected	- neuron-glia communication (especially with astrocytes)<br>- OPC migration	NO, suggestive speculation
Lutz et al 2017	MDD | Suicide | Child abuse	Anterior cingulate cortex (Brodmann 24 and Brodmann 32)<br>*Douglas-Bell Canada Brain Bank*	Genome.wide DNA methylation<br>Histology / Stereology<br>Bulk RNA-seq	= PDGFRα+ cells density<br>↓SOX-10+ cells density in MDD-CA vs. CON and MDD (white matter)	In MDD-CA:<br>- Methylation changes in oligodendrocytes<br>- Transcriptional changes in myelin-related pathways<br>↓ Axonal diameter in MDD-CA<br>↓ Myelin content in MDD-CA	The investigated pathways were mostly unaffected in neurons	NO
Windrem et al 2017	SCZ	Glia progenitor cells (hGPCs) derived from SCZ and CON and transplanted in *Shiverer* mice	RNAseq<br>qPCR<br>Western blot<br>Histology	Aberrant migration pattern (lower hGPCs in white matter and premature hGPCs entry in the gray matter)<br>↓OLIG1<br>↓OLIG2<br>↓SOX10<br>↓GPR17	↓MBP immunostaining<br>↓Myelin luminance<br>↓TF	- Delay in astrocyte maturation (less complex morphology)<br>- Downregulation of synaptic markers	Suggestive: Impaired glia-glia communication (OL lineage and astrocytes)
McPhie et al 2018	SCZ	- Human OL differentiated from IPSCs<br>- White matter of the right prefrontal cortex (MTR)<br>- Whole brain	Single Cell RNAseq<br>Immunofluorescence<br>Magnetization Transfer Ratio (MTR) and T1-weighted<br>Exome Sequencing	↓$O4^{+}$ cells	↓$O4^{+}$ cells	NO	NO

		segmentation (T1-weighted)					
Tanti et al 2018	MDD | Suicide | Child abuse	Ventromedial prefrontal white matter (adjacent BA11, BA12 and BA32)<br>*Suicide section of the Douglas-Bell Canada Brain Bank (Douglas Institute, Montreal, Canada)*	Histology<br>Immunoblotting	=PDGFRα^{+} cells density<br>= SOX10^{+}/NOGOA^{-}<br>↓OLIG2^{+} cells in MDD-CA<br>↓Olig2^{+}/APC^{-} cells in MDD-CA<br>OLIG2^{+} cells density (but no protein level) show positive relationship with age in MDD-CA	↑NOGO-A^{+} cell density<br>↑CC1^{+} cell density<br>NOGO-A+ cells density and MASH1 protein level show negative relationship with age in MDD-CA<br>↓OLIG2 protein level<br>↑MASH1 protein level<br>↓MBP protein level in MDD-CA and MDD<br>↑SOX10^{+}Low/NOGO-A^{+} cell density (mature OL)<br>↓SOX10^{+}High/NOGO-A^{+} cell density (newly mature OL)<br>↑OLIG2^{+}Low/CC1^{+} cell density (mature OL)<br>↓OLIG2^{+}High/CC1^{+} cell density (newly mature OL)<br>= MOG, MAG, CNP, PLP	NO	NO

de Vrij et al 2019	SCZ	Peripheral Blood DNA<br>Human iPSC-derived OPCs<br>Whole brain (DTI)<br><br>*Non-consanguineous family of Dutch ancestry*<br>*Independent Dutch SCZ & Rotterdam Study cohort*	Illumina HumanCytoSNP-12v2 chip arrays<br>(Linkage and copy number analysis)<br>TaqMan Genotyping & Sanger Sequencing<br>Myelination assay on oeganitypic *Shiverer* slices<br>Magnetic Resonance Imaging (MRI) - DTI	In CSPG4A131T carrier iPSCs-derived OPCs:<br>- Aberrant NG2 protein folding<br>- Abnormal NG2 protein sub-cellular localizazion<br>- Decreased ratio NG2 (>300kDA) and NG2 (<300kDa)<br>- Smaller OPCs<br>- Lower cell viability<br><br>In healthy iPSC-derived OPCs transfected with CSPG4V901G :<br>- NG2 protein accumulation in intracellualr vesiclesy<br>- Lower cell viability	In CSPG4A131T carrier iPSCs-derived OPCs:<br>- Reduced maturation<br>- Reduced downstream myelination<br><br>MRI - DTI:<br>- Higher number of white matter potholes<br>- Reduced global FA	NO	NO, only reference to non-canonical pathway
Papiol et al 2019	SCZ	Hippocampus (in vivo MRI)<br>DNA from blood<br><br>*Department of Psychiatry and Psychotherapy of the University Medical Center Goettingen*	SNPs Genotyping<br><br>MRI T1-weighted magnetization- prepared rapid gradient echo (MP-RAGE)	OPC-polygenic risk score (PRS) is associated with changes in CA4/DG volume in response to high intensity aerobic exercise.	Mature OL-PRS does not show association with changes in hippocampal volume in response to high intensity aerobic exercise.	Radial glia-polygenic risk score (PRS) is associated with changes in CA4/DG volume in response to high intensity aerobic exercise.	NO, only reference to non-canonical pathway.
Vasistha et al 2019	Major Mental Illnesses (MMIs)	iPSC-derived OPCs<br>Whole-brain MRI<br><br>*Reported extended Scottish family*	iPSC-derived OPCs<br>qRT-PCR<br>RNAseq<br>diffusion MRI (dMRI), T1-weighted	↓PDGFRα+ cells 7 days post-plating (case 3, case 4)<br>↓ Proliferation (case 3, case 4)	- ↓structural connectivity & white matter integrity<br>- ↑O4⁺ cells at 3-weeks differentation (case 3, case 4)<br>- Mature OL complexity (sholl analysis)<br>- Dysregulation of myelin- and OL differentiation-related mRNA<br>- Alteration in myelin internode formation (mouse chimera)	= GFAP⁺ cells density<br>= TUJ1⁺ cells density<br>↓full-length *DISC1* mRNA	NO

Kolomeetss et al 220	MDD	Putamen<br>*SMRI Neuropathology Consortium Collection*	Histology	↓OPC density in males<br>= OPC denstiy in females	= Mature OL in males and females	NA	NO
Kolomeetss et al 220	BD	Putamen<br>*SMRI Neuropathology Consortium Collection*	Histology	↓OPC density in males<br>= OPCsdenstiy in females	= Mature OL in males and females	NA	NO
Kolomeetss et al 220	SCZ	Putamen<br>*SMRI Neuropathology Consortium Collection*	Histology	↓OPC density in males<br>= OPC denstiy in females	↓ Mature OL in males<br>= Mature OL in females	NA	NO
Nagy et al 2020	MDD | Suicide	Brodmann area 9<br>*Douglas–Bell Canada Brain Bank*	Single-nucleus transcriptomics<br>RNAscope<br>High-throughput PCR	High transcript dysregulation<br>↑*KAZN* in OPCs<br>↓*HSP90AA1* in OPCs	NA	- High transcript dysregulation in deep layer excitatory neurons<br>- 90 ligand-receptor interaction including OPCs and excitatory neurons were changed<br>- ↓ FIBP in deep layer excitatory neurons<br>- Transcriptome changes in interneurons and astrocytes	YES:<br>- OPC-excitatory neuron communication<br>- Stress hormone receptor cycling<br>- other neuronal and non-neruonal cell types<br>- FGF-related pathway
Di Biase et al 2022	SCZ	34 cortical regions (MR)<br>DNA (SNPs genotyping)<br><br>*(1) Human Connectome Project-Young Adult Sample*<br>*(2) Human Connectome Project for Early Psychosis*<br>*(3) Australian Schizophrenia Research Bank*<br><br>*Allen Brain Institute*	T1-weighted MRI<br>SNPs Genotyping<br>Gene-expression	Genetic load related to OPCs associates with less severe cortical thinning	Genetic load related to glial cells associates with less severe cortical thinning	Genetic load related to glial cells associates with less severe cortical thinning<br>Genetic load related to neuronal cells associates with widespread cortical thinning	NO

Kokkosis et al 2022	MDD | Suicide	Brodmann area 9<br>*Douglas–Bell Canada Brain Bank*	Single-nucleus transcriptomics<br>(usage of already published data)	Alteration in oligodendrocyte progeny	Alteration in myelin-related transcripts<br>Reductions of the OL numbers	Immune oligodendrocytes<br>Alteration in cell fate commitment:<br>(1) Immune-OL→ OLs population,<br>(2) Immune-OL→pre-Ols population	NO
Tanti et al 2022	MDD | Child abuse	Brodmann area 11/12<br>*Douglas–Bell Canada Brain Bank*<br><br>Brodmann area 9<br>*Douglas–Bell Canada Brain Bank*	Immunostaining<br>In situ hybridization<br>Single-nucleus transcriptomics	PNN-related transcripts in OPCs correlated with PNN density<br>↑ PNN-related transcripts in OPCs in MD-CA<br>PNN density correlate with OPC-PV neuron proximity and the<br>↑ OPC-PV neuron proximity in MD-CA.<br>= PDGFRA$^+$ cells density (ISH)	NA	↑PNN intensity and density/complexity in CA<br>↑PV surrounded by PNN	YES:<br>- Formation/regulation of PNN by OPCs<br>- PV interneuron and OPC communication
Wingo et al 2022	PTSD	Dorsolateral prefrontal cortex (proteomics)<br>dPFC, frontal cortex, temporal cortex, inferior frontal gyrus, superior temporal gyrus, perirhinal gyrus.<br>*European descent of the Religious Orders Study and Rush memory project*<br>*Banner Sun Health Research Insitute*<br>*Mount Sinai Brain Bank*	Transcriptome-wide association study (TWAS)<br>Proteom-wide association study (PWAS)	Genes enriched in OPCs are (causally) implicated in PTSD	NA	Genes enriched in excitatory neurons are (causally) implicated in PTSD	NO

Yu et al 2022	SCZ	Cortex, hippocampus, amygdala<br>*National Health and Disease Human Brain Tissue Resource Center at Zhejiang University in China*	Immunohistochemistry	↑number of OPC branches<br>= OPC density<br>DISC1 is highly expressed in OPCs<br>↑DISC1 - delta3 and delta7 variants	Only at preclinical level (increased truncated DISC1):<br>↓ oligodendrocyte differentiation<br>↓ myelinated axons	↑WNT/BETA-CATENIN signalling, i.e. RNF43 and WIF1 intensity<br><br>At preclinical level (increased truncated DISC1):<br>↓ number of excitatory synapses<br>↑OPC-(defective) neuron contact<br>↑WNT/BETA-CATENIN signalling	YES, partially demonstrated by preclinical model:<br>- Alteration in Wnt/beta-catenin signalling<br>- Reduction of excitatory synapses
Maitra et al 2023	MDD	Brodmann area 9<br>*Douglas–Bell Canada Brain Bank*	Single-nucleus transcriptomics	↓Number of OPCs<br>OPCs mostly affected in males	NA	In males, implication of astrocytes and excitatory neurons.<br>In females, implication of microglia and PV-interneurons.	NO, only reference to non-canonical pathway.
Yu et al 2023	Multiple disorders	Whole-brain CT<br>Cognitive screening<br>Samples for SNPs screening (e.g. blood, hair…)<br>*ABCD Study (Release 3.0, November 2020)*	MRI Scan<br>Psychological tests<br>Genome-wide association study	OPC-related genetic load associated with changes in the thickness of the left caudal middle frontal gyrus, specifically in internalising disorders	NA	- GABAergic neurons-related genetic load associated with changes in the thickness of the left caudal middle frontal gyrus, specifically in internalising disorders<br><br>- astrocytes-related genetic load associated with changes in cortical thickness, specifically in internalising disorders	NO
Zhou et al 2023	MDD | Suicide	Brodmann area 9<br><br>Brodmann area 11, Brodmann area 25<br>*Multiple Dataset & Douglas Bell Canada Brain Bank*	Differential Gene Expression (DGE)<br>Differential Methylated Regions (DMR)<br>Cross-omics correlation analysis<br>Functional annotation	OPC-related transcript and methylation are enriched in MDD-suicide	Methylation changes in oligodendrocytes	Alteration in ion channels<br>Alteration in glutamate receptors	YES, speculative | discussion section:<br>Changes in ion channels and glutamate receptros specifically in OPCs

Aranda et al 2024	SCZ	Dorsolateral prefrontal cortex<br>*6 different collections of the PsychENCODE project: BrainGVEX, BrainSpan, CMC, BipSeq, LIBD and CMC-HBCC*	Bulk RNAseq<br>(usage of already published data)	- *DDR1* transcripts are enriched in OPCs<br>- ↓ sub-groups of DDR1 transcripts in OPCs (related to morphology during cell cycle	- ↓ sub-groups of *DDR1* transcripts in Oligodendrocyets (related to morphology during cell cycle	*DDR1* related/expressed in other cell types:<br>- Astrocytes - cell morphology during cell cycle<br>- Inhibitory neurons - stablisation of synapses	NO
Aranda et al 2024	BD	Dorsolateral prefrontal cortex<br>*6 different collections of the PsychENCODE project: BrainGVEX, BrainSpan, CMC, BipSeq, LIBD and CMC-HBCC*	Bulk RNAseq<br>(usage of already published data)	- *DDR1* transcripts are enriched in OPCs<br>- ↓ sub-groups of *DDR1* transcripts in OPCs (related to morphology during cell cycle	- ↓ sub-groups of *DDR1* transcripts in Oligodendrocyets (related to morphology during cell cycle	*DDR1* related/expressed in other cell types:<br>- Astrocytes - cell morphology during cell cycle<br>- Inhibitory neurons - stablisation of synapses	NO
Xie et al 2024	MDD	Brodmann area 9<br>*Douglas–Bell Canada Brain Bank*	Single-nucleus transcriptomics	OPC-gene clusters highly predictive of the accurence of MDD	*MALAT1* and *DLG2* gene expressed in OPCs and mature OL and highly associated with MDD	NO	NO, only references to non-canonical pathway.

Supplementary table 3. Summary of the included control studies: method and implicated cell types.

Mental Illness	Method	Tissue of interest	Sample size	Most represented cell type	Reference
Alchol use disorder	snRNA-seq (Brenner E et al 2020)	Prefrontal cortex	AUD, n=3<br>CON, n = 4	Somatostatin neurons<br>Layer 5 extratelencephalic neurons	Joshi A et al 2024
Alchol use disorder	snRNA-seq	Prefrontal cortex	AUD, n=3<br>CON, n = 4	Astrocyte<br>Oligodendrocyte<br>Microglia	Brenner E et al 2020
Bipolar Disorder	**Multiomic approach**<br>scRNA-seq data - PsychENCODE Consortium (Synapse ID: syn7067037)<br>Bulk RNA-seq data (Synapse ID: syn3270015)<br>GWAS summary data (Mullins et al, 2021)<br>ATAC-seq data (Synapse ID: syn7349497)	Whole Brain	**ATAC-seq** | BD, n = 25; CON, n = 185<br>**bulk RNA-seq** |BD, n = 69; CON, n = 245<br>**GWAS** | BD, n = 1,917, CON, n = 371,549	Astrocytes<br>Microglia<br>**<u>OPCs</u>**	Wei W et al 2024
Bipolar Disorder	scRNA-seq dataset<br>GWAS	Brain (10 brain regions)	BD, n = 158,036<br>CON, n = 2,796,499	Medium Spiny Neurons<br>Interneurons<br>Hippocampal pyramidal neurons	O'Cornnell KS et al 2025
Cocaine use disorder	snRNA-seq	Ventral striatum	20,759 single nuclei<br>CUD, n=16<br>CON, n = 8	Medium Spiny Neurons (D1 and D2)<br>Astrocytes<br>(OPCs)	Zillich E et al 2025
Major Depressive Disorder	snRNA-seq	Dorsolateral prefrontal cortex (BA9)	MDD-sucide, n=17<br>CON, n=17	**<u>OPCs</u>**<br>Excitatory neurons	Nagy C et al 2020

Major Depressive Disorder	snRNA-seq (GSE144136 - Nagy C et al 2020)	Dorsolateral prefrontal cortex (BA9)	MDD-sucide, n=17<br>CON, n=17	Endothelial cells<br>Astrocytes	Lian K et al 2025
Major Depressive Disorder	snRNA-seq (GSE144136 - Nagy C et al 2020)	Dorsolateral prefrontal cortex (BA9)	MDD-sucide, n=17<br>CON, n=14	Excitatory neurons	Li X-Y et al 2025
Major Depressive Disorder	scRNA-seq (GSE213982 - Maitra et al 2023)	Dorsolateral prefrontal cortex (BA9)	MDD-suicide, n = 20<br>CON, n = 18	Astrocytes	Pan Y et al 2024
Major Depressive Disorder	snRNA-seq (GSE144136, Nagy et al 2020)	Dorsolateral prefrontal cortex (BA9)	MDD-sucide, n=17<br>CON, n=17	**<u>OPCs</u>**	Xie P et al 2024
Major Depressive Disorder	snRNA-seq (GSE144136, Nagy et al 2020)	Dorsolateral prefrontal cortex (BA9)	MDD, n = 34<br>CON, n = 37	Astrocytes (male MDD)<br>**<u>OPCs (male MDD)</u>**<br>excitatory neurons (male MDD)<br>Parvalbumin interneuron (female MDD)<br>Microglia (female MDD)	Maitra M et al 2023
Major Depressive Disorder	Integration with published dataset (Gandal et al 2018) and GWAS	Cortex		Somatostatin neurons<br>Astrocytes	Anderson KM et al 2020
Major Depressive Disorder (symptoms)	snRNA-seq<br>Integration of GWAS studies	Dorsolateral prefrontal cortex	Old individuals, n = 424	Excitatory neurons<br>Inhibitory neurons	Zeng L et al 2024

Major Depressive Disorder<br>Post Traumatic Stress Disorder	scRNA-seq	Dorsolateral prefrontal cortex (BA9)	MDD, n = 52<br>PTSD, n = 16<br>CON, n = 50<br><br>Batch1: 362,996 nuclei<br>Batch2: 137,230 nuclei	**PTSD:** Excitatory neuron (88% DEG), inhibitory neurons (19% DEG) and astrocytes (3% DEG).<br><br>**MDD:** Astrocyte (48% DEG), excitatory neurons (27% DEG), inhibitory neurons (25% DEG), Oligodendrocyte (4% DEG)<br>**<u>OPCs (1% DEG)</u>**<br>Endothelial cells (<1% DEG)	Daskalakis NP et al 2024
MDD<br>PTSD	snRNA-seq	Dorsolateral prefrontal cortex	MDD, n = 10<br>PTSD, n = 11<br>CON, n = 11	Excitatory neurons<br>Inhibitory neurons<br>Astrocytes	Chatzinakos C et al 2023
Neuropsychiatric disorders	snRNA-seq	Dorsolateral prefrontal cortex	MDD/PTSD, n = 10<br>SCZ, n = 77<br>BD, n = 34<br>CON, n = 182	Excitatory neurons (SCZ, BD)<br>Microglia (SCZ, BD)<br>Oligodendrocyte (BD)	Emani PS et al 2024
Neuropsychiatric disorders	scRNA-seq (PsychENCODE)	Dorsolateral prefrontal cortex		Deep cortical layer excitatory neurons (SCZ)<br>Glia (PTSD, MDD)<br>Vascular cells (PTSD, MDD)	Huuki-Myers LA et al 2024
Neuropsychiatric disorders	**Multiomic approach**<br>snRNA-seq<br>ATAC-seq<br>Integration of GWAS studies	Anterior frontal lobe	CASES, n = 9<br>CON, n = 3	Astrocytes (Obsessive compulsive disorder)<br>**<u>OPCs (anxiety and anorexia, MDD and SCZ)</u>**<br>Multiple neuronal subtypes (MDD, BD, SCZ)	Zhu K et al 2023

Opioid use disorder	snRNA-seq	Striatum	OUD, n = 6<br>CON, n = 6	Microglia<br>Endothelial cells<br>Interneurons<br>Dopaminergic neurons (D1/D2 hybrid)	Phan BN et al 2024
Schizophrenia	rnRNA-seq<br>(Analysis PsychENCODE publicly available data)	Prefrontal cortex	SCZ, n=12M/12F)<br>CON, n=12M/12F<br>(>400,000 single nuclei)	Astrocytes	Zhou R et al. 2025
Schizophrenia	**Multiomic approach**<br>scRNA-seq data - PsychENCODE Consortium (Synapse ID: syn7067037)<br>Bulk RNA-seq data (Synapse ID: syn3270015)<br>GWAS summary data (Pardinas et al, 2018)<br>ATAC-seq data (Synapse ID: syn7349497)	Whole Brain	**ATAC-seq** | SCZ, n = 135; CON, n = 137<br>**bulk RNA-seq** |SCZ, n = 559; CON, n = 936<br>**GWAS** | SCZ, n = 40,675, CON, n = 64,643	Microglia	Cheng B et al 2025
Schizophrenia	snRNA-seq	Prefrontal cortex (BA46)	SCZ, n = 94<br>CON, n = 97	Astrocytes<br>Glutamatergic (excitatory) neurons<br>GABAergic (inhibitory) neurons	Ling E et al 2024
Schizophrenia	snRNA-seq (3 fetuses)<br>Integration with data on genetic variance related to SCZ (Trubetskoy V et al 2022)	frontal cortex, ganglionic eminence, hippocampus, thalamus, and cerebellum	SCZ, n = 76,755<br>CON, n = 243,649	Glutamatergic neurons	Cameron D et al 2022

Schizophrenia	snRNA-seq dataset<br>(https://gtexportal.org/home/datasets)<br>Integration of GWAS studies	Prefrontal cortex<br>Hippocampus	**European**<br>SCZ, n = 40,675<br>CON, n = 64,643<br><br>**Asian**<br>SCZ, n = 22,778<br>CON, n = 35,362	Excitatory neurons<br>Medium spiny neurons<br>GABAergic neurons	Akingbuwa WA et al 2022
Schizophrenia	scRNA-seq dataset	Neocortex, hippocampus, hypothalamus, striatum and midbrain		Hippocampal CA1 pyramidal cells<br>Striatal MSNs<br>Neocortical<br>Somatosensory pyramidal cells<br>Cortical interneurons	Skene NG et al 2018
Schizophrenia/schizoaffective<br>Bipolar Disorder	snRNA-seq<br>Integration with published dataset (Gandal et al 2018) and GWAS	Dorsolateral prefrontal cortex<br>Subgenual cortex		**Transcripts**<br>Astrocytes (SCZ)<br>Microglia (SCZ, BD)<br>Endothelial cells (SCZ, BD)<br>Excitatory and inhibitory neurons (BD)<br><br>**GWAS**<br>**OPCs (BD, MDD)**<br>Neurons (BD, MDD)	Kim B et al 2023
Schizophrenia/schizoaffective<br>Bipolar Disorder<br>Major Depressive Disorder	snRNA-seq	Orbitofrontal cortex (BA11)	Postmortem brain, n=92<br>snRNA-seq ~787,046<br>CASES, n=57<br>CON, n=35	Excitatory neurons<br>Glia<br>Endothelial cells	Gerstner N et al. 2025
